# Self-association and subcellular localization of Puumala hantavirus envelope proteins

**DOI:** 10.1038/s41598-018-36879-y

**Published:** 2019-01-24

**Authors:** Hannah Sabeth Sperber, Robert-William Welke, Roberto Arturo Petazzi, Ronny Bergmann, Matthias Schade, Yechiel Shai, Salvatore Chiantia, Andreas Herrmann, Roland Schwarzer

**Affiliations:** 1 Institute for Biology, IRI Life Science, Humboldt-Universität zu Berlin, Invalidenstr. 42, 10115 Berlin, Germany; 2 Vitalant Research Institute, 270 Masonic Ave, San Francisco, CA 94118 USA; 3 University of Potsdam, Institute of Biochemistry and Biology, Karl-Liebknecht-Str. 24-25, 14476 Potsdam, Germany; 4 Department of Biomolecular Sciences, Weizmann Institute of Science, Rehovot, Israel; 5 Gladstone Institute of Virology and Immunology, 1650 Owens Street, San Francisco, CA 95158 USA

## Abstract

Hantavirus assembly and budding are governed by the surface glycoproteins Gn and Gc. In this study, we investigated the glycoproteins of Puumala, the most abundant Hantavirus species in Europe, using fluorescently labeled wild-type constructs and cytoplasmic tail (CT) mutants. We analyzed their intracellular distribution, co-localization and oligomerization, applying comprehensive live, single-cell fluorescence techniques, including confocal microscopy, imaging flow cytometry, anisotropy imaging and Number&Brightness analysis. We demonstrate that Gc is significantly enriched in the Golgi apparatus in absence of other viral components, while Gn is mainly restricted to the endoplasmic reticulum (ER). Importantly, upon co-expression both glycoproteins were found in the Golgi apparatus. Furthermore, we show that an intact CT of Gc is necessary for efficient Golgi localization, while the CT of Gn influences protein stability. Finally, we found that Gn assembles into higher-order homo-oligomers, mainly dimers and tetramers, in the ER while Gc was present as mixture of monomers and dimers within the Golgi apparatus. Our findings suggest that PUUV Gc is the driving factor of the targeting of Gc and Gn to the Golgi region, while Gn possesses a significantly stronger self-association potential.

## Introduction

Hantaviruses (HVs) pose a global health threat, infecting ca. 30000 individuals each year worldwide^1^. They are considered emerging pathogens which, due to changing climate conditions and therefore redistribution of their natural hosts, have the potential to cause major outbreaks in human populations^2^. Depending on the HV species, infections can lead to HV cardiopulmonary syndrome (HCPS) or hemorrhagic fever with renal syndrome (HFRS)^2–4^. HCPS is caused by New World HVs, and occurs in America, whereas HFRS origins from Old World HVs, found in Europe and Asia^5–7^. Most infections in Europe are caused by the Old World species Puumala virus (PUUV)^8^.

All HVs harbor a trisegmented, negative sensed, single stranded RNA genome. The medium size vRNA segment (M-vRNA) encodes for two glycoproteins, Gn and Gc, which together form the spike complexes of the viral envelope^9^. Gn and Gc play key roles in virus entry, assembly and budding^10,11^. Unlike most enveloped viruses, HVs lack a matrix protein and it has been proposed that the cytoplasmic tails (CTs) of the glycoproteins, especially that of Gn, functions as matrix protein surrogate^10,11^. Through its interaction with the viral nucleocapsid protein and the vRNA segments, Gn mediates the assembly and budding of infectious virus particles^12–17^. Where this process takes place is still under debate^11^, but the Golgi complex is generally believed to be the major budding site of HVs^18–20^. However, for some species, assembly and budding may occur at the plasma membrane instead^21,22^.

**Figure 1:**
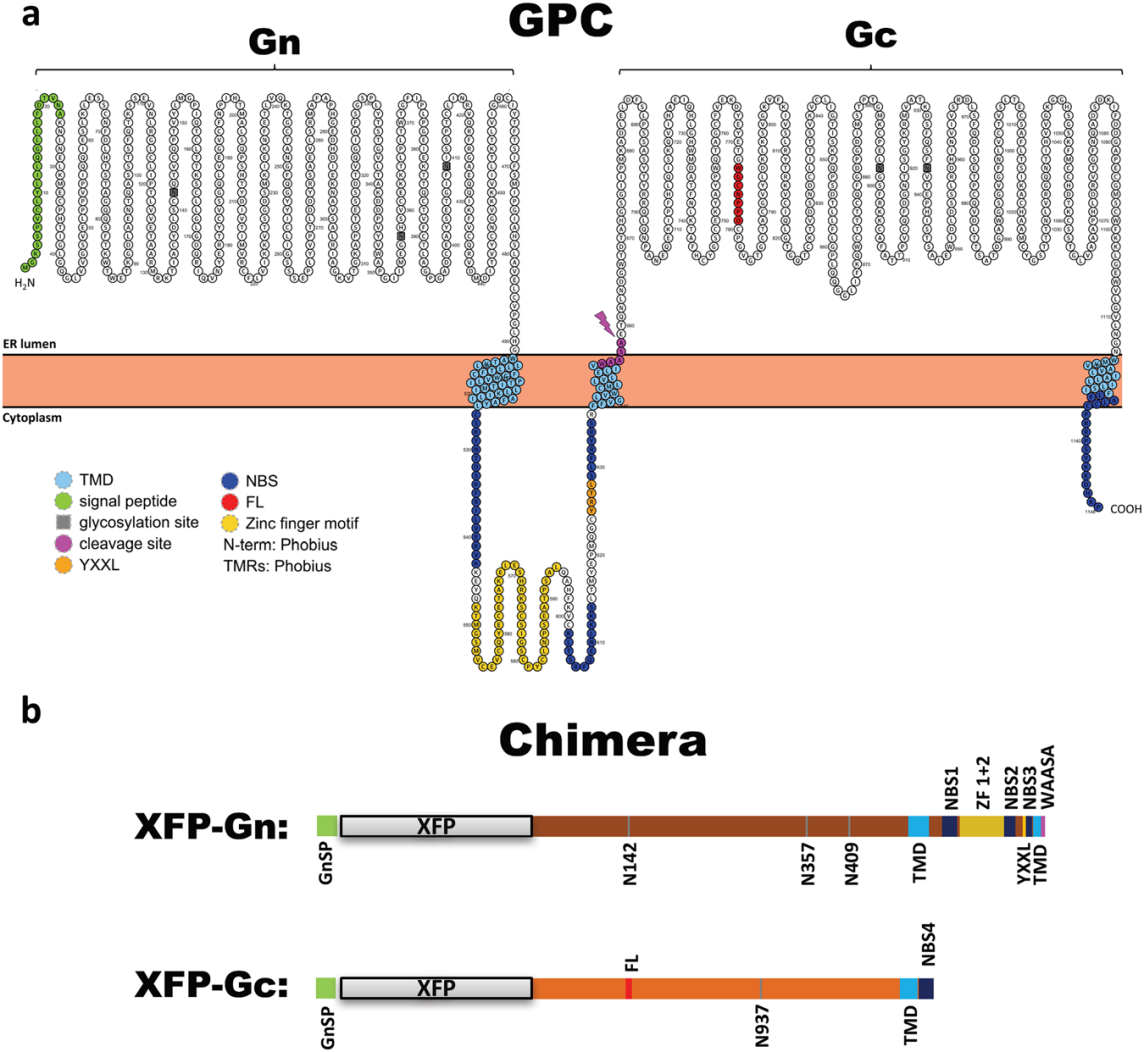
Topological and structural organization of HV glycoproteins Gn and Gc, emerging from the glycoprotein precursor GPC. (**a**) Shown is the aa sequence of the PUUV glycoprotein (UniProt: P27312) from the Sotkamo strain (V-2969/81). The GPC SP is displayed in green (aa 1–24), glycosylation sites in grey, the putative TMDs in light blue (Gn: aa 485–590 and aa 637–656, Gc: aa 1108–1129), the N-protein binding sites in dark blue (NBS1: aa 526–543, NBS2: aa 602–614, NBS3: aa 629–635, NBS4: aa 1131–1148), the zinc finger motifs in yellow (aa 548–594), a YXXL ITAM motif in orange (aa 625–628), the intraprotein cleavage site - WAASA - in pink (aa 654–658), and the fusion loop FL - WGCNPPD in red (aa 773–779). The figure was generated with Protter^75^, motifs were obtained from Andes virus (ANDV) data^11^ and assigned to the PUUV sequence by string alignment between UniProt sequences from ANDV (Q80DP7) and PUUV (Q9WJ31) GPC. Transmembrane sequences were determined by in silico prediction using Phobius^76^. (**b**) Structural elements of the chimeric PUUV glycoproteins created in this study. The highlighted domains are described above. Aa positions and size of the individual domains are drawn to scale. Depending on the experimental requirements, XFP represents a monomeric yellow fluorescent protein (mYFP) or turquoise fluorescent protein (Turq).

The first 24 amino acids (aa) of the glycoprotein precursor (GPC) encode for a signal peptide (SP)^23^, which targets the nascent GPC to the ER membrane^11^. After synthesis of GPC^23^, Gn and Gc emerge as independent proteins in the endoplasmic reticulum (ER) due to a co-translational cleavage at the highly conserved WAASA motif ^24^. Following cleavage, Gn and Gc remain anchored to the ER membrane by their transmembrane domains (TMDs), with their N-terminal ectodomains protruding into the ER lumen and the shorter C-terminal moieties facing the cytoplasm (Fig. 1a). In recent years, different functional motifs in both glycoproteins have been identified and characterized, including N-protein binding site^13,16^, zinc finger motifs^25,26^, ITAM motif^27^, and fusion loop^28^.

The mature HV spike complex consists of four Gn and four Gc subunits and displays a four-fold rotational symmetry^29–31^. So far, the genesis of the spike complex has been only insufficiently characterized and novel studies of other members of the *Bunyaviridae* family revealed a surprisingly high diversity in the organization of the spike complexes^31–35^.

In this work, we generated fluorescent, chimeric protein variants of the two PUUV glycoproteins Gn and Gc for separate expression in mammalian cells. This strategy enabled investigating specific properties of the individual viral proteins in absence of other viral components and allowed for the identification of specific functional motifs. By integrating the SP of GPC (GnSP) in the open reading frame of both proteins, we enabled a correct and physiological targeting of their translation, a factor that may have been disregarded in previous studies. We performed localization studies, utilizing fluorescence microscopy and quantitative image analysis, showing that Gc translocates to the Golgi apparatus in absence of other viral components. In contrast, we found that Gn localizes in the Golgi region only upon co-expression with Gc. Of note, co-expression led to a significant increase in Golgi localization of both proteins, demonstrating a mutual enhancement of their Golgi targeting. By site-directed mutagenesis and C-terminal truncations, we identified the GcCT to be necessary, but not sufficient for Golgi localization of the viral glycoproteins. Finally, by assessing the oligomerization of the chimeric proteins via fluorescence anisotropy imaging microscopy (FAIM) and Number&Brightness (N&B) analysis, we demonstrate that Gn possesses a significantly higher tendency to oligomerize than Gc, supporting previous data that suggests that Gn may form the tetrameric spikes on the hantavirus surface^16,36^. Taken together, our results indicate that Gn and Gc have complementary biological activities that enable a tightly controlled organization of the intracellular targeting of the glycoproteins and the assembly of the homomeric subunits.

## Results

### Construction of N-terminal labeled chimeric PUUV glycoproteins

The fluorescent tag was attached to the N-termini of the PUUV glycoproteins (Fig. 1b) to minimize any interference with putative interactions of cytoplasmic factors that could be required for glycoprotein trafficking and localization, since several important interaction sites and functional motifs have been identified within the CTs of Bunyavirus glycoproteins^13,16,27,37,38^. Particularly in the case of Gc, which possesses a very short CT, a bulky label being linked to the C-terminus of the protein would impact the biological activity of this important protein domain.

### mYFP-Gc cloning strategy

The N-terminal signal peptide is targeting the nascent precursor protein GPC to the ER, thus determining the ER as synthesis site of both emerging glycoproteins. Taking this into consideration, both of our glycoprotein variants were designed to contain the GnSP attached to the N-terminus of the respective chimeric construct (Fig. 1b). Therefore, the GnSP is translated first and then co-translationally cleaved from the following chimeric protein, comprised of a fluorescent protein (XFP) and the respective glycoprotein sequence. This cloning strategy (including a SP at the N-terminus of both proteins) is required to ensure proper targeting of the expression and thus subcellular localization for both constructs and should reproduce a physiological expression of HV glycoproteins.

### mYFP-Gn and mYFP-Gc differ in their intracellular localization when expressed independently

First, we investigated the intracellular distribution of the constructs by co-localization analysis with markers for cellular compartments that are putatively involved in the PUUV life cycle: ER, Golgi apparatus and plasma membrane (PM). To enable live-cell imaging we co-transfected mYFP-Gn or mYFP-Gc with the marker proteins pmTurquoise2-Golgi (Golgi-Turq, see SI Fig. 1) and a glycosylphosphatidylinositol-anchored cyan FP (GPI-CFP) to stain Golgi apparatus and PM, respectively. The ER was stained with ER-tracker Red prior to live-cell microscopy.

**Figure 2:**
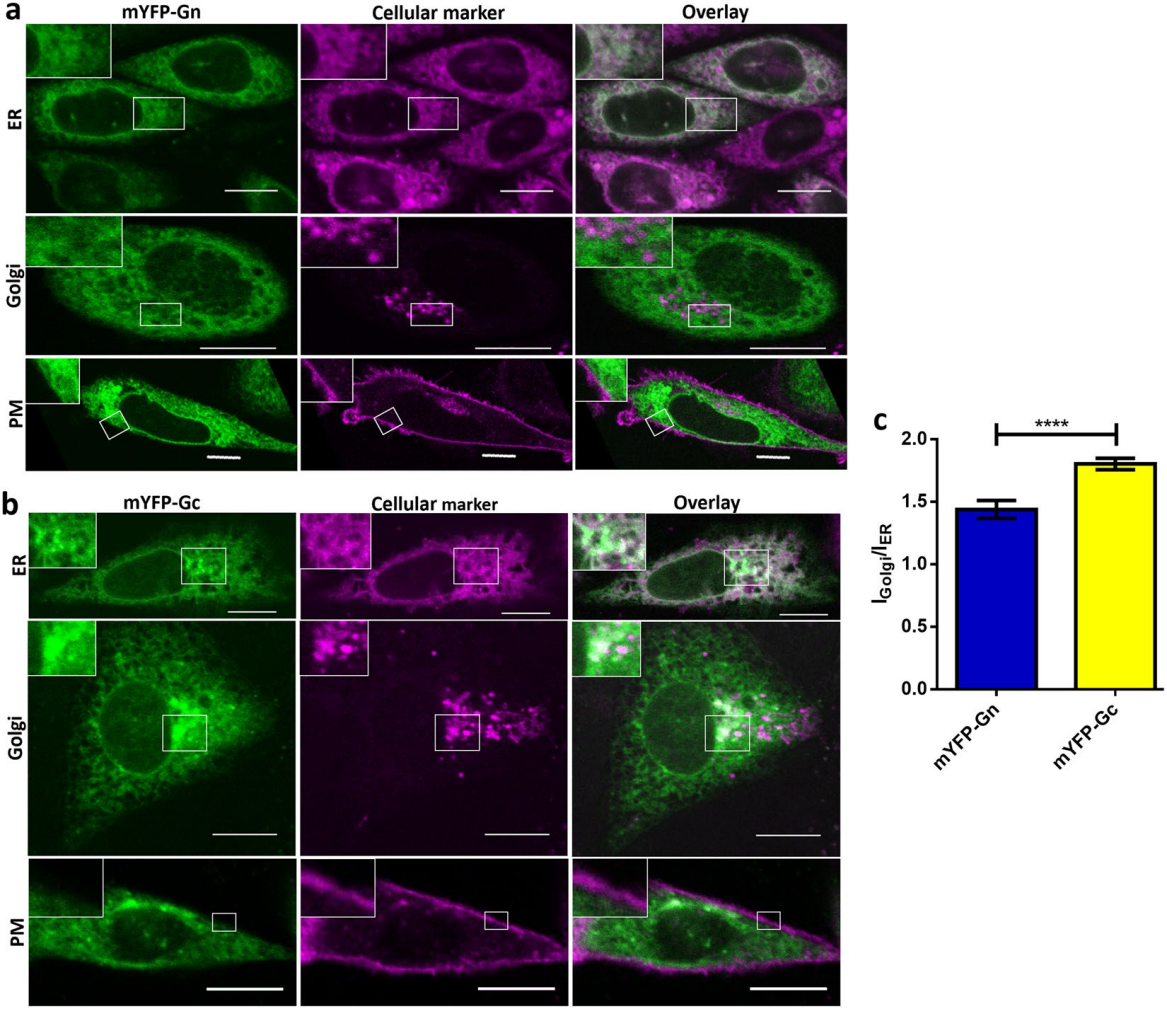
Intracellular localization of separately expressed PUUV chimeric glycoproteins mYFP-Gn and mYFP-Gc. CHO-K1 cells were co-transfected with either (**a**) mYFP-Gn or (**b**) mYFP-Gc and different cellular markers of the Golgi apparatus (Golgi, marker: Golgi-Turq) or plasma membrane (PM, marker: GPI-CFP), respectively. The endoplasmic reticulum (ER) was stained after transfection using ER-tracker Red. Fluorescence images represent equatorial slices of cells and were obtained by confocal microscopy 24 hours post transfection. Insets show magnifications of the boxed regions. High degrees of co-localization appear white in overlay images. Scale bar = 10 µm. (**c**) Quantification of mYFP-Gn and mYFP-Gc enrichment in the Golgi apparatus by ImageJ analysis. The Golgi region was identified based on the compartment marker Golgi-Turq. The columns show the mean of 28 cells expressing mYFP-Gn and 60 cells expressing mYFP-Gc from at least four independent experiments. Error bars show the standard error of the mean (SEM) of individually analyzed cells. The asterisks denote ****p ≤ 0.0001 by Student’s test for unpaired data.

When expressed seperately, both glycoproteins strongly co-localized with the ER marker but not with GPI-CFP at the PM (Fig. 2a,b). Interestingly, we found a significant co-localization of mYFP-Gc with the Golgi-marker (Fig. 2b), whereas mYFP-Gn appeared to be excluded from the Golgi apparatus (Fig. 2a).

To quantify our visual observation, we assessed co-localization of Golgi-Turq and the chimeric glycoproteins using ImageJ (Fig. 2c). Determined pixel intensities in the Golgi and ER region were expressed as the ratiometric value (I_Golgi_/I_ER_), which directly reflects Golgi accumulation of the protein under study. This analysis revealed a highly significant enrichment of mYFP-Gc in the Golgi apparatus compared to mYFP-Gn (Fig. 2c), thus confirming our previous observation. To test whether this finding resulted from differences in the kinetics of mYFP-Gn and mYFP-Gc protein synthesis and trafficking, we analyzed different time points post transfection (SI Fig. 2). However, we found comparable intracellular distributions for both proteins at 12, 24 and 48 hours post transfection, indicating that the protein localization is in a steady-state already after 12 hours and suggesting that differences in the subcellular localization indeed reflect distinct trafficking properties of the two viral glycoproteins. We also expressed both proteins in two additional cell types, Human embryonic kidney 293 cells (HEK-293 cells) or African green monkey kidney epithelial cells (Vero E6 cells), in order to investigate cell and species dependency of the protein properties. In both cell lines, the intracellular localization of mYFP-Gn and mYFP-Gc again differed greatly, similar to CHO-K1 cells, with a strong perinuclear enrichment of mYFP-Gc and a broader distribution of mYFP-Gn (SI Fig. 3).

### Co-expression of mYFP-Gn and Turq-Gc leads to a significant enrichment of both proteins in the Golgi apparatus

**Figure 3:**
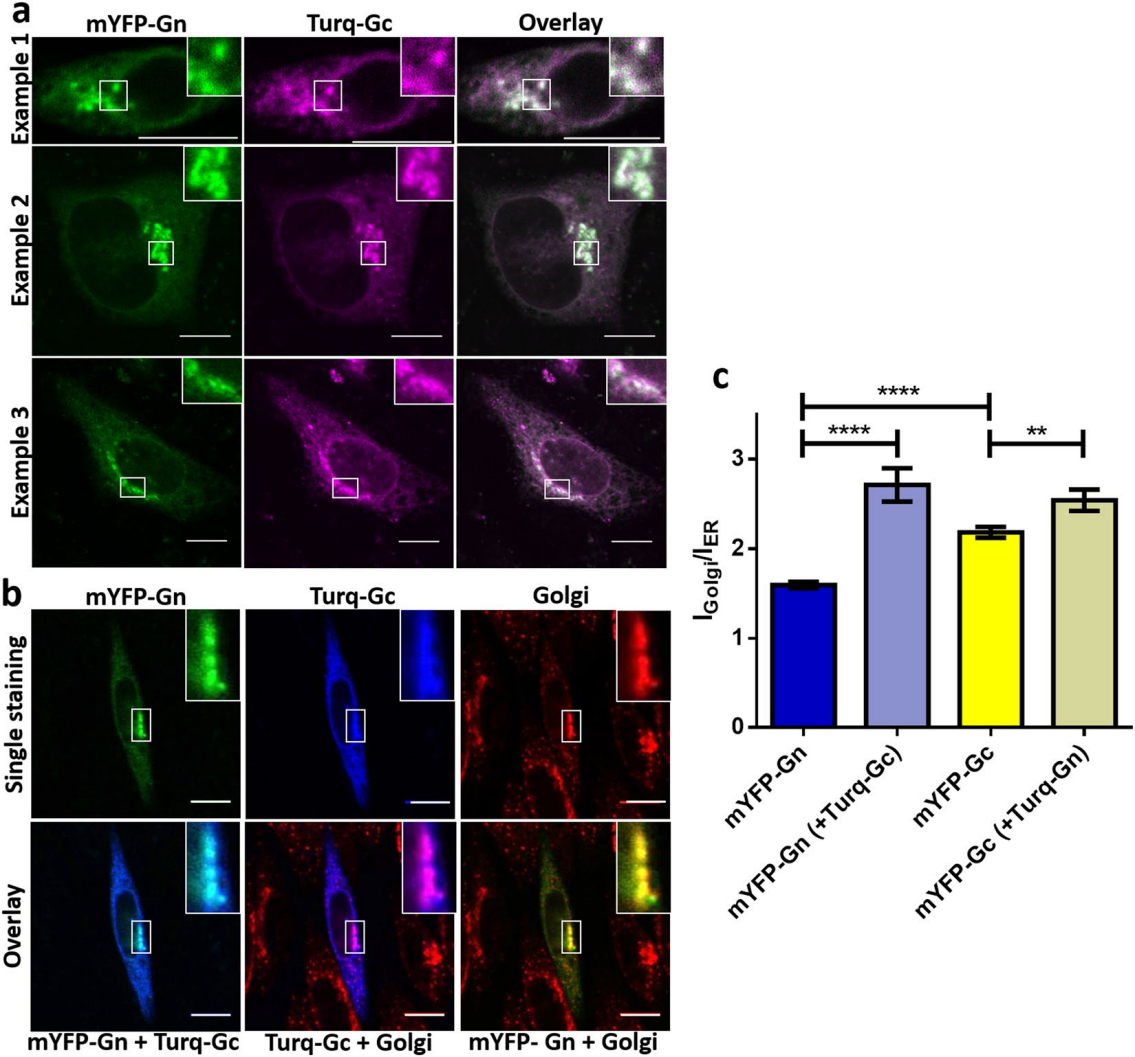
Intracellular distribution of co-expressed PUUV glycoproteins in living CHO-K1 cells. (**a**) Co-transfection of mYFP-Gn (green) and Turq-Gc (purple). Samples were analyzed 24 hours post transfection. Three typical cells are displayed. White areas indicate co-localization. (**b**) Immunofluorescence staining of CHO-K1 cells, co-transfected with mYFP-Gn (green) and Turq-Gc (blue), utilizing anti-membrin-Alexa647 as marker of the Golgi-apparatus (red). Insets show magnifications of the boxed regions. Scale bar = 10 µm. (**c**) Quantification of mYFP-Gn and mYFP-Gc enrichment in the Golgi apparatus upon co-expression using ImageJ. The Golgi apparatus was identified based on anti-membrin staining in fixed cells. Columns show the mean of 36 cells expressing mYFP-Gn only, 23 cells expressing mYFP-Gn and Turq-Gc, 35 cells expressing mYFP-Gc only, and 27 cells expressing mYFP-Gc and Turq-Gn, from three independent experiments. Brackets indicate Turq-tagged proteins. Error bars show SEM of individually analyzed cells. The asterisks denote ****p ≤ 0.0001 and **p ≤ 0.01 by Student**’**s test for unpaired data.

Next, we studied the intracellular distribution of mYFP-Gn and Turq-Gc upon co-transfection. In co-expressing cells, Turq-Gc emerged in the ER and Golgi apparatus resembling the subcellular distribution of mYFP-Gc in absence of mYFP-Gn (compare Figs 2b and 3). In contrast, the intracellular localization of mYFP-Gn changed markedly (Fig. 3). Whereas in absence of Turq-Gc, mYFP-Gn was mainly localized in the ER (Fig. 2), it strongly accumulated in the perinuclear region upon co-expression, showing strong co-localization with Turq-Gc (Fig. 3a). To verify the identity of this accumulation site, we additionally stained the cells after co-transfection with an anti-membrin antibody^39,40^ as a reporter of endogenous Golgi proteins. Both glycoproteins clearly co-localized with membrin (Fig. 3b), thus confirming that mYFP-Gn and Turq-Gc accumulate in the Golgi apparatus.

Image quantification revealed a significant increase in the Golgi accumulation for both glycoproteins in co-expressing cells (Fig. 3c), suggesting that co-expression is promoting ER exit and Golgi targeting of mYFP-Gn and Turq-Gc, and thus enabling efficient delivery of both spike components to the putative site of PUUV assembly and budding.

### The full-length GcCT is necessary for a proper Golgi localization of mYFP-Gc

**Figure 4:**
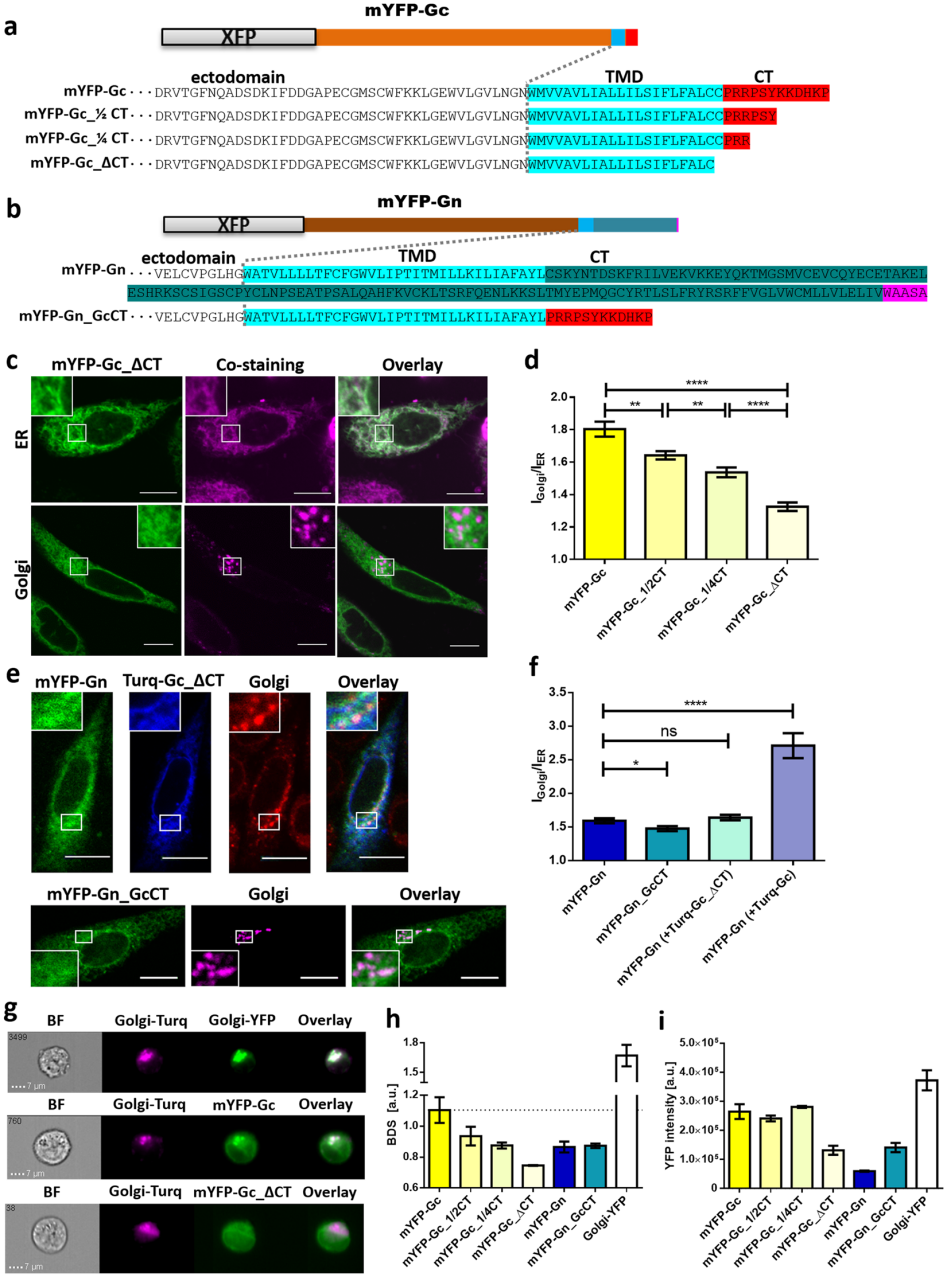
Intracellular localization of mYFP-GcCT and mYFP-GnCT variants. (a) + (b) Schematic representation of GnCT and GcCT variants. (**a**) The CT of mYFP-Gc is represented in red, (**b**) the CT of mYFP-Gn is represented in dark green, the WAASA sequence is shown in pink. TMD = transmembrane domain (light blue). (**c**) Intracellular localization of mYFP-Gc_ΔCT (green), co-stained with the ER tracker Red (upper panel) or co-transfected with Golgi-Turq (lower panel). Co-localization appears white in overlay images. (**d**) Quantification of glycoprotein co-localization with Golgi-Turq using ImageJ. Columns show the mean of 60 cells expressing mYFP-Gc, 65 cells expressing mYFP-Gc_½CT, 46 cells expressing mYFP-Gc_¼CT and 70 cells expressing mYFP-Gc_ΔCT from at least four independent experiments. (**e**) The upper panel displays immunofluorescence staining of fixed cells that were co-transfected with mYFP-Gn (green) and Turq-Gc_ΔCT (blue) utilizing anti-membrin-Alexa647 as a Golgi-marker (red). The lower panel shows co-transfection of mYFP-Gn_GcCT (green) and Golgi-Turq (purple). Insets show magnifications of the boxed regions. (**f**) Quantification of the Golgi enrichment of the mYFP-Gn and mYFP-Gn_GcCT in presence and absence of the Gc variants Turq-Gc and Turq-Gc_ΔCT. The Golgi apparatus was identified based on anti-membrin staining (in fixed cells) or Golgi-Turq co-expression. The YFP mean fluorescence ratio I_Golgi_/I_ER_ was determined from 36 cells expressing mYFP-Gn, 24 cells expressing mYFP-Gn_GcCT, 28 cells expressing mYFP-Gn and Turq-Gc_ΔCT, and 23 cells expressing mYFP-Gn and Turq-Gc, from three independent experiments. Brackets indicate Turq-tagged proteins. (**g**–**i**) Imaging flow cytometry analysis of glycoproteins co-transfected with Golgi-Turq. (**g**) Representative cells co-expressing Golgi-Turq (in pink) and different constructs (in green). Golgi-YFP, mYFP-Gc and mYFP-Gc_ΔCT are shown as examples for strong, moderate and low co-localization with Golgi-Turq. (**h**) Quantitative co-localization analysis of Golgi-Turq with different chimeric proteins, expressed as bright detail similarity (BDS). (**i**) Expression analysis of chimeric proteins by assessing single-cell fluorescence intensities from at least three independent experiments. In imaging flow cytometry experiments, a minimum of 2000 cells were analyzed per transfection sample. Error bars show SEM from independent experiments. Asterisks denote: ****p ≤ 0.0001, ***p ≤ 0.001, **p ≤ 0.01, *p ≤ 0.05, p > 0.05 (ns) by Student**’**s test for unpaired data. For statistics of BDS (h) and YFP intensity (i) see SI Table 1 and SI Table 2, respectively. Scale bars = 10 µm. All experiments were performed 24 hours post transfection.

In a next step, we focused on mYFP-Gc and its ability to accumulate in the Golgi. We hypothesized that the CT is responsible for Golgi enrichment due to its exposure to cytoplasmic cellular factors, which may promote mYFP-Gc transport. We therefore generated chimeric Gc variants that featured different truncations of the CT (Fig. 4a) and quantified Golgi localization of each construct by ImageJ analysis. The new protein variants, mYFP-Gc_½CT, mYFP-Gc_¼CT and mYFP-Gc_ΔCT lack half, three quarters or the entire CT, respectively.

mYFP-Gc_ΔCT displayed a negligible co-localization with the Golgi-, and a high co-localization with the ER-marker (Fig. 4c). This observation was confirmed by quantitative image analysis, revealing a highly significant decrease in Golgi localization of mYFP-Gc upon truncation of its CT (Fig. 4d: mYFP-Gc vs. mYFP-Gc_∆CT). Of note, already the modest truncation of the CT, introduced in the variant mYFP-Gc_½CT, resulted in a significantly decreased Golgi localization (Fig. 4d: mYFP-Gc vs. mYFP-Gc_½CT, SI Fig. 4). This reduction was even more pronounced for mYFP-Gc_¼CT (SI Fig. 4). Both however, mYFP-Gc_½CT and mYFP-Gc_¼CT, co-localized to a significantly higher degree with the Golgi apparatus than mYFP-Gc_∆CT (Fig. 4d). Altogether, our data revealed a successive reduction of the Golgi-localization upon stepwise truncation of the CT, suggesting that the entire CT is engaged in Golgi targeting rather than specific residues or motifs within the CT.

To extend and verify our confocal microscopy experiments, we applied imaging flow cytometry, a technique that enables high throughput, fluorescence microscopy and automated single-cell analysis (Fig. 4g–i). Using this approach, we evaluated and compared the intracellular localization of our glycoprotein constructs with respect to the Golgi-marker Golgi-Turq, based on their bright detail similarity (BDS) (Fig. 4h). The BDS function correlates the small bright image detail of two images and can be used to quantify the co-localization of two fluorescently labeled proteins. The BDS results were consistent with our data obtained by image analysis (compare Fig. 4d,f with Fig. 4h). Again, the full length mYFP-Gc was found to strongly co-localize with the Golgi apparatus and this property decreased upon truncation of its CT (Fig. 4h). Furthermore, mYFP-Gn was significantly less associated with the Golgi complex compared to mYFP-Gc (Fig. 4h, SI Table 1).

### Co-expression of mYFP-Gn and Turq-Gc_∆CT does not rescue Golgi localization

We next investigated the effect of eliminating the entire CT of our chimeric Gc on Golgi targeting function in the context of glycoprotein co-expression and asked whether mYFP-Gn can rescue Turq-Gc_ΔCT trafficking defects. Golgi staining of cells co-transfected with mYFP-Gn and Turq-Gc_ΔCT revealed no enrichment of mYFP-Gn or Turq-Gc_ΔCT in the Golgi region, and both constructs exhibited intracellular patterns as observed when expressed separately. (compare Figs 2a and 4e, and Fig. 4c,e). This result, corroborated by our quantitative analysis (Fig. 4f), again emphasized the importance of an intact GcCT for a proper intracellular distribution of the chimeric Gn and Gc constructs.

### GcCT is not sufficient for Golgi targeting

Our previous results suggested that GcCT may be necessary for an efficient Golgi accumulation of both PUUV glycoproteins. To test if the GcCT alone is sufficient to permit Golgi targeting, we replaced the CT of Gn by GcCT (Fig. 4b, mYFP-Gn_GcCT) and investigated mYFP-Gn_GcCT by confocal microscopy and imaging flow cytometry. Both assays showed that substituting the GnCT with the GcCT did not lead to a pronounced accumulation of mYFP-Gn_GcCT in the Golgi complex. mYFP-Gn_GcCT remained in the ER, displaying a similar distribution pattern as observed for the original mYFP-Gn construct (Figs 2a and 4e,f,h).

### GnCT but not GcCT influences protein expression

We analyzed the overall fluorescence intensities of the YFP-tagged proteins using imaging flow cytometry to assess the expression level of the different YFP-tagged Gn and Gc variants. Our data indicated that the successive truncation of the GcCT did not considerably alter the expression of mYFP-Gc, reflected by similar YFP intensities for the glycoprotein variants mYFP-Gc, mYFP-Gc_½CT, and mYFP-Gc_¼CT (Fig. 4i, SI Table 2). In contrast, mYFP-Gc_∆CT exhibited a significant reduction in protein expression, presumably a result of an impaired stability of the truncated protein, an increased likelihood of folding defects and premature degradation of the protein.

We consistently observed a significantly lower fluorescence intensity for mYFP-Gn than for mYFP-Gc, a finding that may indicate differences in the kinetics of synthesis and/or degradation. Interestingly, an increased protein expression was assessed for mYFP-Gn_GcCT compared to mYFP-Gn, even though we did not detect an accumulation of mYFP-Gn_GcCT in the Golgi region (Fig. 4h,i). This implies that mYFP-Gn_GcCT either exhibits a higher protein stability or a more efficient expression than the original mYFP-Gn variant.

### mYFP-Gc and mYFP-Gn are exposed at the cell surface and GcCT truncations increase PM localization

**Figure 5:**
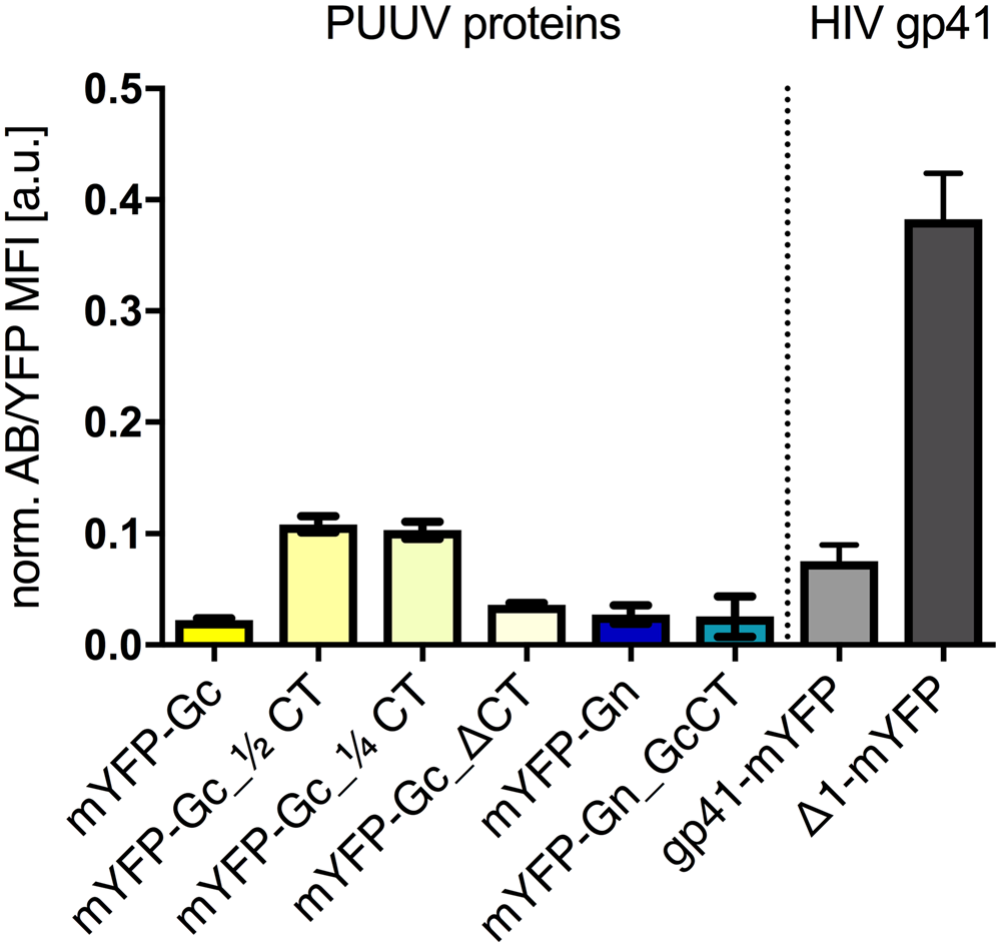
Surface exposure analysis by flow cytometry. Cells transfected for 24 hours were stained with anti-GFP fluorescent antibodies and the mean fluorescence intensity of the antibody was assessed as a reporter of PM exposure. Samples were normalized to the positive control GPI-mYFP. Two previously published HIV gp41 fusion proteins were analyzed for comparison. Error bars show the SEM of independent experiments. For statistics see SI Table 3.

We analyzed the PM localization of YFP-tagged PUUV glycoproteins by flow cytometry. To this aim, CHO-K1 cells, transfected with different protein constructs were stained with anti-GFP antibodies (being cross-reactive to all variants of Aequorea victoria GFP) to specifically label surface exposed, chimeric proteins. We used GPI-mYFP, a known Golgi apparatus and PM marker, as a positive control, normalizing all proteins under study to this construct. We observed a detectable antibody signal for all constructs (Fig. 5), whereas for mock transfected cells the signal was virtually undetectable (not shown). This finding clearly demonstrates surface exposure of the glycoprotein constructs. However, in contrast to the positive control GPI-mYFP (SI Table 3), the signals were significantly lower, supporting our previous observation of an only minor PM exposure of the PUUV glycoproteins (Figs 2 and 5, SI Table 3). Interestingly, all GcCT truncation variants were weakly, but significantly enriched at the PM compared to mYFP-Gc, suggesting that the 6 C-terminal amino acids of mYFP-Gc may be involved in a regulated restriction of mYFP-Gc surface exposure. Of note, mYFP-Gc_ΔCT showed significantly lower surface expression than the other two truncation variants, mYFP-Gc_½CT, and mYFP-Gc_¼CT. This may be, as suggested before, a result of an impaired stability of the truncated protein, which supposedly increases misfolding and leads to partial ER retention and degradation. We also analyzed two previously published variants of the human immunodeficiency virus (HIV) glycoprotein gp41^41^. As expected, gp41 showed a weak surface exposure (relative to GPI-mYFP) that is significantly increased upon removal of the protein’s CT (∆1-mYFP), containing several known endocytosis motifs.

### mYFP-Gn exhibits a higher degree of oligomerization than mYFP-Gc

**Figure 6:**
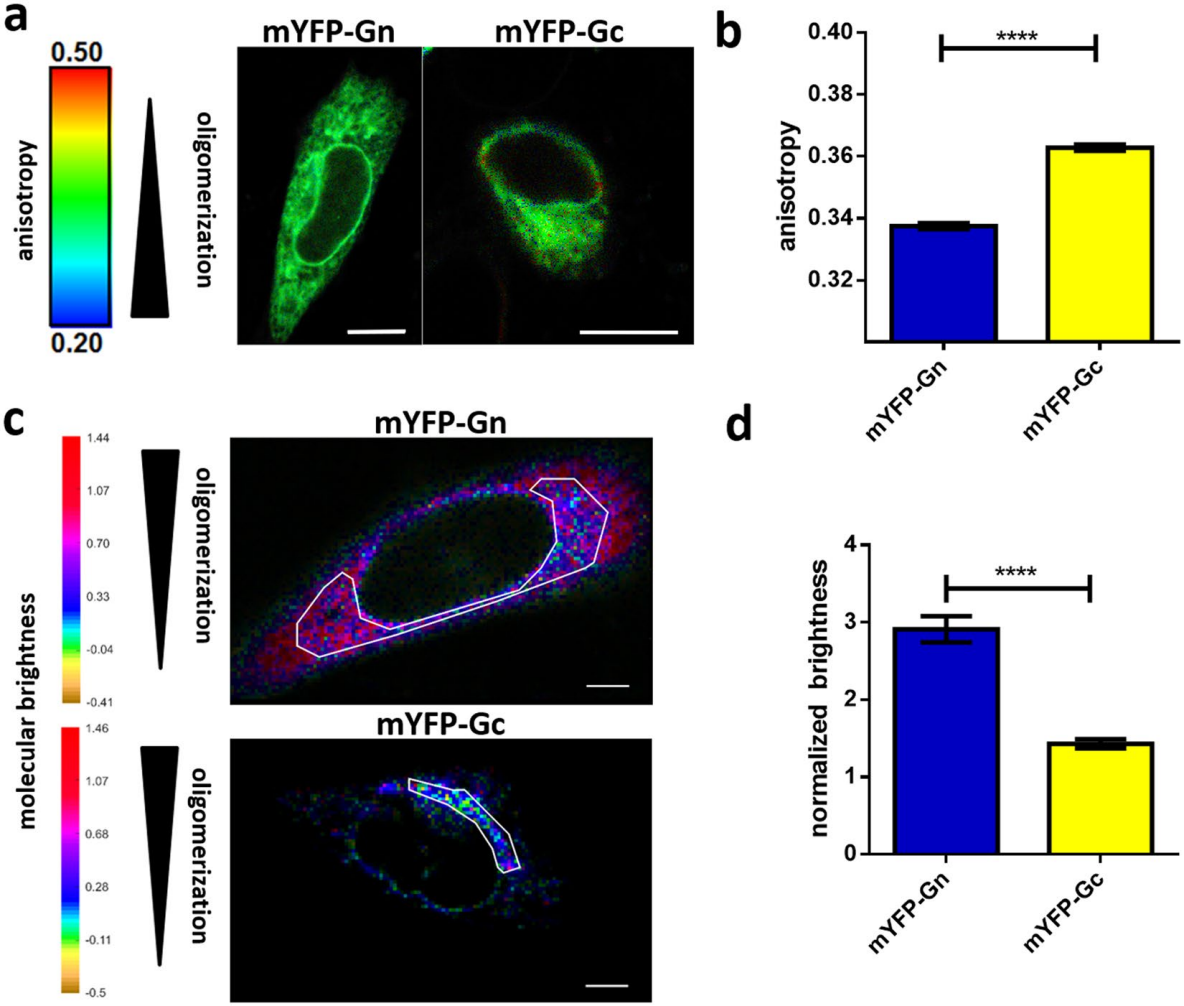
Oligomerization of PUUV glycoproteins expressed in living CHO-K1 cells studied via fluorescence anisotropy and brightness mapping. (**a**) CHO-K1 cells were transfected with mYFP-Gn or mYFP-Gc and investigated by FAIM. The pseudo-colored images display the degree of oligomerization of the respective chimera in each cell based on the measured fluorescence anisotropy. (**b**) Bars show the mean, single cell anisotropy values of 52 cells expressing mYFP-Gn and 96 cells expressing mYFP-Gc from at least four independent experiments per specimen. Error bars show the standard error of the mean. The significance was assessed by Student’s test for unpaired date and is displayed by asterisks: ****p ≤ 0.0001. (**c**) Brightness maps of typical CHO-K1 cells expressing mYFP-Gn or mYFP-Gc, respectively. The color bar shows the brightness scale expressed as photon counts per molecule per 100 µs (pixel dwell time). Scale bar = 5 µm. (**d**) Brightness normalized using the corresponding monomer value extracted from the analysis of mYFP-ER or mYFP-Golgi (see main text for details). For each cell, an ROI enclosing the ER or the Golgi was defined and analyzed. The graph shows the mean values of the normalized brightness measured over 25–40 cells transfected with YFP-tagged fusion proteins from at least three independent experiments. Error bars represent the SEM. The asterisks denote ****p ≤ 0.001 as defined by MATLAB**’**s ttest2 function for unpaired data.

To investigate the self-association properties of PUUV glycoproteins, we applied fluorescence anisotropy imaging microscopy (FAIM) (Fig. 6a,b). This method enables a reliable and rapid detection of homo-FRET - Förster resonance energy transfer between interacting proteins that harbor identical fluorophores^42^ - and was used before to investigate self-clustering and oligomerization of fluorescently labeled proteins^41,42^. In this assay, self-association/oligomerization is reported by a decrease of the fluorescence anisotropy values due to homo-FRET. Our experiments show that mYFP-Gn expressing cells exhibited significantly lower YFP anisotropy values than mYFP-Gc expressing cells (Fig. 6b), indicating a higher tendency of mYFP-Gn to self-associate and suggesting that Gn oligomerizes in absence of other viral factors.

### mYFP-Gn assembles into dimers and tetramers in the ER

To quantitatively assess the oligomerization state of mYFP-Gn and mYFP-Gc, we additionally performed N&B analysis in a live, single-cell format. This technique allows quantification of the oligomerization state of fluorescent molecules by determining their molecular brightness. Through comparison to the brightness of fluorescent monomer and dimer references (i.e. by calculating a normalized brightness), N&B directly provides the average amount of fluorescent proteins in a complex that is diffusing within a cell^43,44^. Fig. 6c shows brightness maps of typical CHO-K1 cells expressing YFP-tagged Gn or Gc, respectively. In this representation, the pixel intensity indicates the average amount of photons per pixel (i.e. fluorescence intensity). The color represents the average brightness of molecules in the pixel, in units of photons per molecule per 100 µs (pixel dwell time). In each of the analyzed maps, a region of interest was chosen corresponding to the ER (for mYFP-Gn), or to the Golgi (for mYFP-Gc). Cellular organelles were identified by co-expression of the markers mCherry-ER and mCherry-Golgi (see SI Fig. 7). Fig. 6d shows the normalized brightness measured in 25–40 cells expressing mYFP-Gn or mYFP-Gc, averaged over all the pixels enclosed in the selected regions of interest. The normalization through mYFP monomer and dimer brightness references implies that the values shown here give a direct measure of the average multimerization state of the glycoprotein constructs in the examined samples (see Materials and Methods). For example, a mixture of monomers and dimers would correspond to a normalized brightness value between 1 and 2 (see equation (4)). Our results indicate that mYFP-Gc forms small oligomers, while mYFP-Gn assembles into larger complexes. Such outcome is in full agreement with the results obtained from anisotropy measurements. To allow for quantitative conclusions regarding the multimerization state of the analyzed constructs, some simplifying assumptions must be made. Assuming that mYFP-Gc is present as a mixture of monomers and dimers within the Golgi complex, the observed normalized brightness of 1.43 ± 0.06 (average ± SEM, n = 28) would indicate a ~73:27 monomer to dimer molar ratio, according to equation (4). Similarly, based on previous biochemistry findings suggesting the organization of Gn in dimers and tetramers^35,44^, the observed normalized brightness value of 2.91 ± 0.17 (average ± SEM, n = 39) for mYFP-Gn would indicate that this protein is present as a mixture of 70 molar% dimers and 30 molar% tetramers (see equation (4)).

## Discussion

Intracellular targeting and assembly of viral proteins is a prerequisite of a successful virus proliferation. In this study, we investigated the subcellular localization and oligomerization of the PUUV glycoproteins Gn and Gc in single, living CHO-K1 cells by using different state-of-the art fluorescence microscopy techniques. The intracellular distribution of both proteins was studied by confocal microscopy and quantitative image analysis. In addition, we performed imaging flow cytometry to verify and expand microscopy-based data, by analyzing thousands of cells in an unbiased, user-independent fashion. For the chimeric PUUV glycoprotein mYFP-Gn, we found a high co-localization with ER markers, but only a minor spatial correlation with Golgi markers. mYFP-Gc, on the other hand, was shown to exhibit a distinct accumulation in the Golgi apparatus. Furthermore, we show that a complete GcCT is required for proper Golgi targeting of mYFP-Gc, since a stepwise truncation of the GcCT led to a corresponding reduction of Golgi localization.

In recent years, several studies attempted to elucidate the intracellular trafficking of HV glycoproteins, mainly using recombinant protein constructs and fluorescence based techniques^17,18,45–49^. Surprisingly, different publications reported partially conflicting results: Pensiero *et al*.^46^ found that Gn of Hantaan virus (HNTV) localizes to the Golgi in absence of other viral components. Shimizu *et al*.^17^ reported a strong co-localization of Gn with the cis-Golgi and a rather diffuse distribution of Gc in the context of a full-length GPC expression. Upon co-transfection with the nucleocapsid protein (NP), Gc was observed to be significantly enriched in the Golgi apparatus. Interestingly, Shimizu *et al*. found that the Golgi-targeting effect on Gc was least pronounced for the PUUV NP, whereas HTNV, Seoul Virus (SEOV) and Sin Nombre Virus (SNV) NP strongly reduced the number of cells with diffused Gc distribution. This may suggest that the targeting of PUUV glycoproteins might be more dependent on intrinsic factors of the PUUV GPC complex. Ruusala *et al*.^47^ and Shi *et al*.^48^ showed that co-expression of HNTV Gn and Gc is required for both proteins to localize to the Golgi apparatus. Similarly, Spiropoulou *et al*.^18^ and Deyde *et al*.^49^ demonstrated for ANDV and SNV, respectively, that co-expression of the glycoproteins is necessary for Golgi localization. Studies focusing on other *Bunyaviridae* family members reported that Gn is capable of localizing to the Golgi complex in absence of Gc or other viral components^50–53^. However, we are the first to demonstrate that a chimeric Gc (and not Gn) of PUUV can be found in the Golgi complex in absence of other viral components. Further, we showed that co-expression of the glycoprotein chimera markedly enhanced Golgi localization of both glycoproteins. In contrast, upon truncation of the GcCT, our chimeric Gn and Gc constructs are excluded from the Golgi. Based on these results, we conclude that Gc is the determining factor for Golgi targeting of both PUUV glycoproteins. Our findings emphasize the interdependency of the two glycoproteins and, regarding intracellular targeting, are in agreement with previous studies, showing most efficient trafficking of both glycoproteins to the Golgi apparatus upon co-expression^18,46–49^. One explanation for a mutual promotion of the Golgi localization of both glycoproteins might be a more efficient translocation of the glycoproteins from the ER to the Golgi apparatus upon formation of mature oligomeric complexes. Another possibility is that the interaction between both glycoproteins prevents premature cellular degradation processes, i.e. autophagosomal degradation (as described for Gn of SNV^54,55^) that might precede intracellular trafficking. However, we could neither detect co-localization of our chimeric Gn construct with autophagosomes nor an enrichment in the Golgi apparatus upon autophagy inhibition (SI Fig. 5). While our findings demonstrated that the GcCT is required for Golgi localization of mYFP-Gc, it was not sufficient to promote Golgi localization of the respective Gn chimera, mYFP-Gn_GcCT. Nevertheless, the significant increase of the expression levels of mYFP-Gn_GcCT compared to mYFP-Gn suggests a role of the CTs in protein expression and/or stability. Indeed, previous publications on NY-1 virus (NY-1V), HNTV, ANDV, TULV, and Prospect Hill virus (PHV) reported that the GnCT is ubiquitinated and involved in subsequent proteasomal degradation^27,37,38^ of the protein. Further studies must determine the role of the CTs for expression and stability of PUUV glycoproteins.

In agreement with previous studies^18,28,56,57^, we show that both proteins, mYFP-Gn and mYFP-Gc, exhibit a detectable degree of plasma membrane expression. However surface exposure was not very pronounced and only minor co-localization with a PM marker was found, which supports earlier publications, reporting primarily intracellular assembly and budding of Hanta- and Bunyaviruses^9,20,46,47,58^. Interestingly, we observed a small, but significant increase in the cell surface exposure of all CT-truncation variants of Gc. This may indicate that the C-terminal section of the GcCT contains a Golgi-retention or re-internalization motif, comparable to the C-terminal dileucine and YSPL motifs that were identified in the HIV-1 glycoprotein gp41^59,60^. In fact, we found comparable increases in surface exposure (4–5 fold) upon partly removal of the CTs of gp41 and Gc (mYFP-Gc vs. mYFP-Gc_¼ CT and gp41-mYFP vs. ∆1-mYFP in Fig. 5, SI Table 3). Interestingly, a previous study on new world HVs demonstrated that GcCT truncations do not affect surface exposure and virus like particle formation in context of ANDV GPC expression^56^. At this point we can only speculate that this difference is indicative of structural and functional distinctions between new world and old world HV glycoproteins that render the PUUV GcCT indispensable for intracellular trafficking whereas the ANDV counterpart can be removed without introducing obvious defects.

We conducted FAIM as well as N&B analysis to study the oligomerization status of mYFP-Gn and mYFP-Gc individually in a live-cell context. Both methods independently revealed a lower degree of self-association for mYFP-Gc than for mYFP-Gn. Moreover, N&B analysis indicated that mYFP-Gc is present as a mixture of mostly monomers and dimers within the Golgi apparatus, whereas mYFP-Gn was found to form mostly dimers and larger oligomers (e.g. tetramers) in the ER. Of note, an investigation of the CT mutants mYFP-Gn_GcCT and mYFP-Gc_∆CT suggested an involvement of GnCT in the protein’s self-association (SI Fig. 6).

Our study is, to the best of our knowledge, the first to investigate HV glycoprotein oligomerization in a live, single cell context. While in recent years the spike complex structure and composition of two Old World HV species, the non-pathogenic Tula virus (TULV)^29^ and pathogenic HNTV^30^, could be unraveled in detail, it remains largely unknown how the tetrameric and mature hetero-oligomeric complexes are initially assembled in the infected cell. Based on our results, we speculate that Gn is the primary trigger and driving factor of spike complex assembly. This would be in line with a model that has been previously formulated, suggesting an initial formation of Gn homo-oligomers (i.e. dimers or tetramers) that is followed by the recruitment of Gc subunits^16,36,61^.

In summary, we report distinct functionalities and biological activities assigned to PUUV glycoproteins: Gc predominantly determines the intracellular trafficking of both glycoproteins and Gn mediates the homo-oligomerization to finally form part of the spike complexes. We want to emphasize that preceding studies investigating different HV species obtained varying results concerning intracellular localization, interaction and oligomerization of HV envelope proteins. This strain specific behavior could be based on a broad evolutionary diversity of HVs which might be a result of the tight co-evolution of HVs and their respective hosts, causing an independent, highly adaptive development of the different HV species. Further studies are necessary to deepen our understanding of this aspect, as well as maturation and functions of the glycoproteins.

## Materials and Methods

### Cloning and generation of chimeric proteins

Puumala virus (PUUV) glycoprotein encoding viral RNA was purified from Vero E6 cells, infected with the PUUV, Sotkamo strain (V-2969/81), an Orthohantavirus from the family of the *Hantaviridae*. After RNA extraction (RNeasy, Qiagen) and reverse transcription, cDNA was used for amplification via PCR. Gc and Gn were independently cloned into pmYFP-N1 vectors including A206K monomeric mutation of the fluorescent tag^62^. At the N-terminus of the fluorophore, the HV glycoprotein signal peptide (SP) was introduced to ensure a physiological membrane incorporation and localization of the fusion protein constructs. The obtained expression plasmids, termed mYFP-Gn and mYFP-Gc, were further modified in our laboratory by site-directed mutagenesis. C-terminal truncations were introduced by classic PCR amplification. Mutations and exchange of the Gn cytoplasmic tail (GnCT) were performed by Quickchange (Stratagene) cloning. All clonings were tested by Sanger sequencing of expression plasmids. pmTurquoise2-Golgi (Golgi-Turq) was a gift from Dorus Gadella (Addgene plasmid #36205) and has been described before^63^. MyrPalm-mYFP (myristoylated and palmitoylated peptide fused to YFP) contains the amino acid sequence MGCIKSKRKDNLNDDEPPVAT derived from the N-terminus of the Lyn kinase. The myristoylation/palmitoylation sequence was then subcloned into pmYFP-N1. mYFP-ER and mYFP-Golgi were obtained from pmTurquoise2-ER (gift from Dorus Gadella, Addgene plasmid #36204) and Golgi-Turq (Addgene plasmid #36205), respectively, by exchange of the fluorophore. For co-expression experiments, chimeric Gc and Gn constructs were generated and fluorescently labeled with Turquoise instead of YFP. Fluorophore exchange was conducted by restriction-based subcloning.

### Cell culture and transfection

Chinese hamster ovary (CHO-K1) cells, Human embryonic kidney 293 cells (HEK-293 cells) or African green monkey kidney epithelial cells (Vero E6 cells) were maintained in Dulbecco’s modified Eagle’s medium (DMEM, PAA Laboratories GmbH, Austria) containing 10% fetal bovine serum. 24 hours prior to experiments, fusion protein expression plasmids were transfected into cell lines in 35 mm-diameter plates utilizing Turbofect (Thermo Scientific, Waltham, MA, USA) according to the manufacturer’s protocol.

### Immunofluorescence staining

If not otherwise stated, cells were subjected to microscopy and flow cytometry without prior fixation. For intracellular immunofluorescence staining however, cells were washed three times with phosphate-buffered saline with calcium and magnesium (PBS+/+) and fixed with 3.7% paraformaldehyde for 25 min at room temperature. Afterwards, the cells were washed three times with PBS+/+ before permeabilization with 0.2% Triton X-100 and 0.2% bovine serum albumin (BSA) for 20 min. After three more washing steps, cells were incubated with anti-membrin antibody (Abcam, ab13511) for 1 h. This procedure was repeated for the AlexaFlour647 conjugated secondary antibody (goat anti mouse IgG, Invitrogen) and the immunostaining was concluded with three washing steps.

### Confocal fluorescence microscopy

Differential interference contrast (DIC) and fluorescence intensity images were obtained with a 60x water immersion objective (numerical aperture 1.2) at 25 °C with a frame size of 512 × 512 pixels. For confocal imaging, CFP or Turquoise were excited at 405 nm using a laser diode and observed in 475–490 nm detection range. YFP was excited at 515 nm using an argon laser and detected in the range of 535–575 nm. AlexaFluor647 was excited at 635 nm using a laser diode and observed in 655–755 nm detection range. Signals of co-expressing cells were recorded sequentially and obtained intensities were analyzed with the ImageJ analysis program.

### Quantitative image analysis

Colocalization of HV proteins with compartment markers was quantified by manual image analysis using ImageJ. Initially, regions of interest (ROIs) were defined, based on the fluorescence signal of fluorescently labeled Golgi marker proteins or fluorescent antibodies (identifying the Golgi apparatus) and DIC images (cell outlines). The ratio I_Golgi_/I_ER_ of mYFP expressing proteins in individual cells was determined by assessing mean mYFP pixel intensity in the Golgi apparatus relative to the mean mYFP pixel intensity in the remaining cell (reflecting the ER).

### Fluorescence anisotropy imaging microscopy (FAIM)

The experimental setup for confocal microscopy is described above. An expanded setup explicitly described on the manufacturer website^64^ was used for fluorescence anisotropy imaging. After removal of DIC depolarization filters, images were obtained with a 60x water objective (numerical aperture 1.2) with a frame size of 512 × 512 pixel. A pulsed 470 nm laser diode with a repetition frequency of 20 MHz was applied to excite mYFP. The polarized emitted light was separated with a polarization beam splitter and parallel and perpendicular fluorescence signals were detected using a 540/40 emission filter for the perpendicular polarized light and a 540/30 emission filter for the parallel polarized light prior to a τ-SPAD and Perkin/Elmer SPAD respectively. The g-factor for this microscope setup was calculated from point scans of the emission signals on both channels with an Alexa488 solution. A value of 1.65 was found for the setup described above. Fluorescence steady state anisotropy pictures were accumulated for 90 s with an average photon count rate of 50,000–100,000 counts per second. Images were analyzed using the SymPhoTime software after selection of suitable regions of interest. The obtained pixel weighted values were summed up into average anisotropy values.

### Statistical test

To address cell-to-cell variance in the parameter under study, for quantitative evaluation of microscopy experiments single cells were analyzed separately. If not otherwise stated, experimental data represent the mean ± SEM of individually analyzed cells. Statistical significance was assessed using a parametric, unpaired, two-tailed Student’s t-test with a 95% confidence interval and significance displayed as follows: ****P < 0.0001; ***P < 0.001; **P = 0.001–0.01; *P = 0.01–0.05.

### Imaging flow cytometry

CHO-K1 cells were first co-transfected with different HV glycoprotein variants and Golgi-Turq for 24 hours, detached using PBS/EDTA (5 mM), washed with PBS without calcium and magnesium (PBS−/−), spun down (200 g, 10 min) and resuspended in PBS−/−. Cells were then subjected to ImageStreamX (Amnis/EMD Millipore) analysis using the IDEAS software (Amnis, EMD millipore)^65^ without previous fixation. Samples were analyzed with a 60x lens, numerical aperture 0.9 generating 40 × 170 µm images with a 330 nm pixel size. Images were compensated for fluorescent dye overlap by using single-stain controls. Cells were gated for single cells or doublets using the area and aspect ratio features, and for focused cells using the GradientRMSfeature as described previously^66^. Co-localization of the Golgi-marker and the protein of interest was determined using the bright detail similarity feature (BDS)^67^. The BDS correlates the small bright image detail of two images and can be used to assess co-localization of two labels. BDS is defined as the log transformed Pearson’s correlation coefficient of localized bright spots with a radius of 3 pixels or less within the masked area in the two analyzed images. The initial correlation coefficient varies between 0 (uncorrelated) and 1 (perfect correlation) and is log transformed to increase the range between zero and infinity (0, inf)^68,69^. In this experimental setup, high values report a strong correlation of the fluorescence signal in both investigated channels (one of which shows a Golgi marker) and therefore an enrichment of the protein of interest in the Golgi-apparatus. Overall glycoprotein expression was assessed by calculating the mean fluorescence intensity of mYFP.

### Flow cytometer membrane expression experiments

CHO-K1 cells were transfected with chimeric proteins for 24 hours, detached using PBS/EDTA (5 mM) and then incubated with AlexaFluor647 anti-GFP antibody (Biolegend, 338005) and Zombie Red (Biolegend, 423109) for 15 min at room temperature. Then, cells were washed, spun down (300 g, 5 min) and resuspended in PBS−/− for flow cytometry analysis. Samples were initially gated for singlets, Zombie negative (viable) populations. Then, mYFP positive cells were gated based on mock transfected samples. The mean fluorescence intensity (MFI) was assessed in the YFP and AlexaFluor647 channel. The MFI in the antibody channel was normalized to the mYFP MFI in order to account for differences in the overall expression of the chimeric proteins for the evaluation of the surface exposure. Finally, the results from all protein variants were normalized to the positive control GPI-mYFP.

### Number and Brightness (N&B)

N&B was performed as previously described^70^. Briefly, CHO-K1 cells were plated onto 35 mm glass-bottom dishes from Ibidi (Ibidi, Planegg, Munich, Germany) 48 h prior to the experiment and transfected utilizing Turbofect (Thermo Scientific, Waltham, MA, USA) according to the manufacturer’s protocol 20–24 h prior to the experiment. Cell imaging was performed at 25 °C. Confocal images were acquired using a Zeiss LSM780 microscope. The 488 nm excitation from a CW Argon laser (Lasos, Jena, Germany) was focused with a 40× UPLS Apocromat 1.2 NA water objective into the sample. The fluorescence signal was collected by a Zeiss QUASAR multichannel GaAsP detector in a 508–597 nm range. Images of 128 × 128 pixels were acquired with pixel dimensions ∼400 nm and a pixel dwell time of 100 μs. Image time-stacks of 100 scans were collected in photon counting mode using the Zeiss Black ZEN software. The intensity time-stacks data were analyzed using self-written Matlab code (The MathWorks, Natick, MA, USA). The Matlab algorithm implements the equations from Digman *et al*.^71^ for the specific case of true photon-counting detectors^72^, thus obtaining the true molecular brightness (referred to simply as ‘brightness’ in what follows) as a function of pixel position. The pixel brightness is a measure of the multimerization state of fluorescent objects diffusing in and out of the pixel. Regions of interest were selected in cell images based on the distribution of specific intracellular markers (i.e. mCherry-ER or mCherry-Golgi, see SI Fig. 7). The selected pixel brightness values were pooled to calculate occurrence histograms. Thereafter, brightness values were normalized in every separate experiment using the brightness value ε_1 of a mYFP monomer localized either in the ER or Golgi (i.e., mYFP-ER or mYFP-Golgi). Since it is not known whether these markers might form dimers, before measuring monomer brightness, cells were subjected to several bleaching cycles with high laser power until the brightness did not decrease anymore. At this point, only monomers or partially bleached dimers (with the same apparent brightness ε_1 of a monomer) should contribute to the measurement.

In order to improve the precision of the brightness calibration, we also took into account the probability of maturation of the fluorescent tag (p_m_)^44,73^. To this aim, it suffices to determine p_m_ for the fluorescent protein of interest in constructs with two known multimerization states and in the typical experimental conditions^44^. This was accomplished by determining the brightness for a membrane bound fluorescent monomer (e.g. MyrPalm-mYFP) or a dimeric tandem (e.g. MyrPalm-mYFP-mYFP, see SI Fig. 8). The latter can be modeled as a mixture of fluorophores with different brightness (${{\rm{\varepsilon }}}_{{\rm{full}}}=2{{\rm{\varepsilon }}}_{1}$ for a fully formed mature dimer; ${{\rm{\varepsilon }}}_{{\rm{partial}}}={{\rm{\varepsilon }}}_{1}$ for a partially fluorescent dimer), with corresponding brightness given by the formula:

1 $${{\rm{\varepsilon }}}_{2}\,({{\rm{n}}}_{{\rm{partial}}},\,{{\rm{n}}}_{{\rm{full}}})=\frac{{{{\rm{\varepsilon }}}_{{\rm{partial}}}}^{2}\cdot {{\rm{n}}}_{{\rm{partial}}}+{{{\rm{\varepsilon }}}_{{\rm{full}}}}^{2}\cdot {{\rm{n}}}_{{\rm{full}}}}{{{\rm{\varepsilon }}}_{{\rm{partial}}}\cdot {{\rm{n}}}_{{\rm{partial}}}+{{\rm{\varepsilon }}}_{{\rm{full}}}\cdot {{\rm{n}}}_{{\rm{full}}}}$$

where ${{\rm{n}}}_{{\rm{partial}}}\,{\rm{and}}\,{{\rm{n}}}_{{\rm{full}}}$ are the numbers of mature dimers and partially mature dimers, respectively.

Assuming that ${{\rm{n}}}_{{\rm{partial}}}\,{\rm{and}}\,{{\rm{n}}}_{{\rm{full}}}$ follow a binomial probability distribution defined by p_m_, the final brightness of a nominal fluorescent dimer with maturation probability p_m_ is given by:

2 $${\varepsilon }_{2}({p}_{m})=({p}_{m}+1)\cdot {\varepsilon }_{1}$$

Note that ${\varepsilon }_{2}=2{\varepsilon }_{1}$, only if *p*_*m*_ = 1.

And in general, for higher multimerization states of the n-th order:

3 $${\varepsilon }_{n}({p}_{m})=((n-1)\cdot {p}_{m}+1)\cdot {\varepsilon }_{1}$$

Measuring the brightness of the dimeric and monomeric controls directly provides the value of p_m_ for mYFP, which is then used to normalize all following measurements. In our experimental conditions, we obtained a p_m_ value of ca. 0.65, as expected for mYFP in general^44^. The value of was measured for each experiment using the above-mentioned ER and Golgi markers (rather than MyrPalm-mYFP), to take into account geometry-related changes in monomer brightness. In conclusion, in all the reported results, a normalized brightness value of 1 corresponds to the brightness *ε*_1_ of a mYFP monomer control; a normalized brightness value of 2 corresponds to the brightness *ε*_2_ of a mYFP dimer control; a normalized brightness of 3 corresponds to the brightness expected for a mYFP trimer *ε*_3_; and so on. This simple correction was shown to provide reliable values up to, at least, 12-mer complexes^44^.

In the case of simple mixtures of two oligomeric species with molar concentrations *n*_*i*_ and *n*_*j*_, with corresponding brightness values *ε*_i_ and *ε*_j_, the measured brightness in a pixel will be:

4 $${\varepsilon }_{mixture}\,({n}_{i},\,{n}_{j})=\frac{{{\varepsilon }_{i}}^{2}\cdot {n}_{i}+{{\varepsilon }_{j}}^{2}\cdot {n}_{j}}{{\varepsilon }_{i}\cdot {n}_{i}+{\varepsilon }_{j}\cdot {n}_{j}}\,$$

Measurements on the Gn and Gc constructs were performed at low excitation intensity. The laser power was set so that the photon count rate remained below 1 MHz (i.e. typical values were ≤4 μW). To correct for residual photobleaching effects and minor cell movements, we used a boxcar-filter pixel-wise with a 8-frame length, as previously described^74^. In our experimental condition, bleaching remained usually below 10–15% of the initial measured intensity. In order to avoid concentration-dependent effects on multimerization determination, all experiments (including those on the monomer and dimer controls) were performed on cells expressing the fluorescent proteins at comparably low concentration levels (i.e. ~10–50 proteins per pixel, corresponding to ~10 nM). Saturation of detectors leading to artefactual reduction in brightness was avoided by excluding pixels in which photon-counting rates exceeded 1 MHz^71^. Using this selection criterion, saturation induced brightness decrease was kept below 10%. Finally, to further correct the weak residual negative correlation between brightness and pixel intensity, we measured the detector response analyzing the signal from a dried Alexa488 solution. The thus obtained brightness vs. intensity plot (which should be constant and equal to 0 for all intensity values, since the fluorophore is immobile) was used to correct the actual experimental data.

## Supplementary information

SI

## Data Availability

The datasets generated during and/or analyzed during the current study are available from the corresponding author on reasonable request.
