# Supplementary material for: Self-association and subcellular localization of Puumala hantavirus envelope proteins
Source: Sci Rep. 2019 Jan 24;9:707. doi: 10.1038/s41598-018-36879-y (PMC6345964; doi:10.1038/s41598-018-36879-y)
Supplement: Supplementary file 1 — SI [file 41598_2018_36879_MOESM1_ESM.docx]

**Self-association and subcellular localization of Puumala hantavirus envelope proteins**

Hannah Sabeth Sperber^1,2^*, Robert-William Welke^1^*, Roberto Arturo Petazzi^3^, Ronny Bergmann^1^, Matthias Schade^1^, Yechiel Shai^4^, Salvatore Chiantia^3^, Andreas Herrmann^1#^, Roland Schwarzer^1,4,5#^

1. Institute for Biology, Humboldt University of Berlin, Invalidenstr. 42, 10115 Berlin, Germany

2. Blood Systems Research Institute, 270 Masonic Ave, San Francisco, CA 94118

3. Institute for Biochemistry and Biology, Potsdam University, Karl-Liebknecht-Str. 24-25, 14476 Potsdam

4. Department of Biological Chemistry, Weizmann Institute of Science, Rehovot, Israel

5. Gladstone Institute of Virology and Immunology, 1650 Owens Street, San Francisco, CA 95158

* shared contribution

# corresponding author

Correspondence and requests for materials should be addressed to R.S. ([Roland-Schwarzer@gmx.de)](mailto:Roland-Schwarzer@gmx.de)) and A.H. ([andreas.herrmann@rz.hu-berlin.de)](mailto:andreas.herrmann@rz.hu-berlin.de)).

# **SUPPLEMENTARY INFORMATION**

**Supplementary Figures**

***Clear co-localization between Anti-membrin antibody and transfected Golgi-marker Golgi-Turq.*** In this study, we made extensive use of the expression plasmid pmTurquoise2-Golgi^1^ (Golgi-Turq) as a Golgi-marker. To verify the specificity of Golgi-Turq, we stained CHO-K1 cells with anti-membrin-A647, targeting the endogenous Golgi residing protein Golgi SNAP receptor complex member 2 (membrin), 24 h after transfection with Golgi-Turq. As expected, we found a strong enrichment of both markers in the Golgi region and also a clear co-localization between them (SI Fig. 1). Thus, Golgi-Turq is qualifed as reference for intracellular localization of the Golgi apparatus and allows for a detailed determination of the cellular distribution of proteins under investigation.


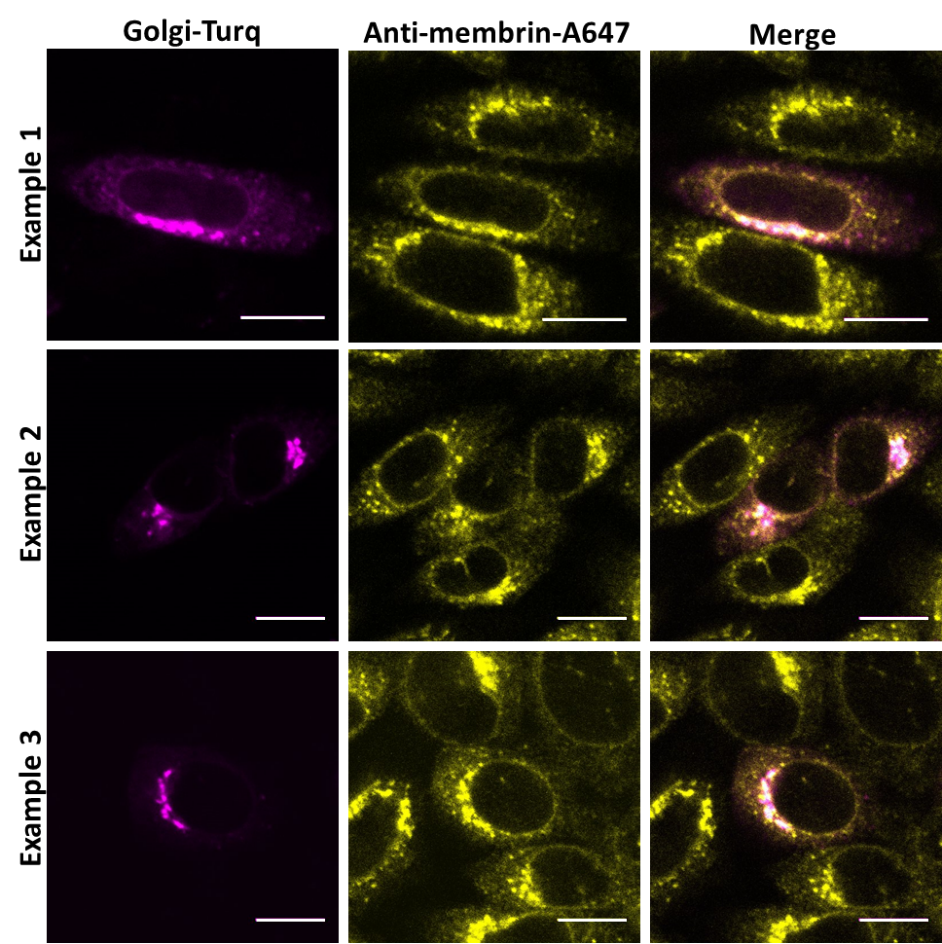


**SI Fig. 1: Anti-membrin-Alexa647 staining of CHO-K1 cells transfected with Golgi-Turq.** Three typical examples are displayed of cells with a stained Golgi apparatus using two different Golgi markers simultaneously. Cells were transfected with the expression plasmid Golgi-Turq (purple) for 24 h. After cell fixation, cells were stained with anti-membrin-Alexa647 (yellow) and subjected to confocal microscopy. Fluorescence images represent equatorial slices. High degrees of co-localization appear white in overlay images. Scale bar = 10 µm.

***Intracellular distribution of mYFP-Gn and mYFP-Gc reflect distinct trafficking patterns of viral glycoproteins.*** We investigated the intracellular localization of our glycoprotein chimera for three different time points (12 h, 24 h, and 48 h) post transfection (SI Fig. 2). Independent of the time points, mYFP-Gn was widely distributed throughout the cell reflecting an ER-like pattern (SI Fig. 2a) while mYFP-Gc was clearly enriched in the Golgi-apparatus (SI Fig. 2b). Thus, the different cellular localizations of mYFP-Gn and mYFP-Gc especially concerning accumulation in the Golgi region are unlikely based on different kinetics of protein synthesis but rather reflect actual distribution patterns of the viral glycoproteins when expressed individually.


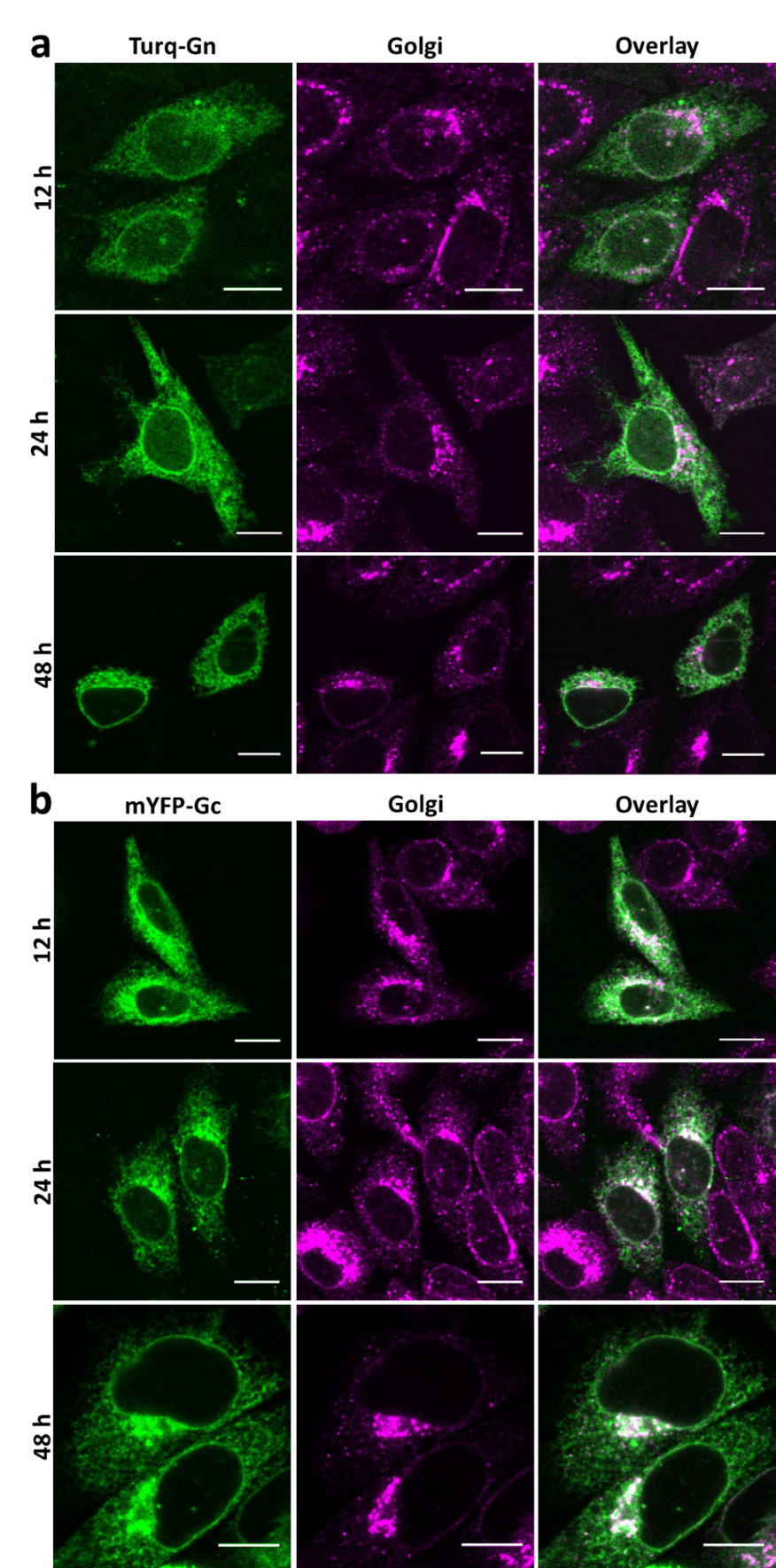


**SI Fig. 2:** **Intracellular localization of chimeric PUUV glycoproteins after different time points of transfection.** CHO-K1 cells were transfected with (a) Turq-Gn (green) or (b) mYFP-Gc (green) for 12 h, 24 h, and 48 h. Fixed cells were stained using anti-membrin-Alexa647 (purple) as marker of the Golgi-apparatus. Fluorescence images represent equatorial slices obtained by confocal microscopy. High degrees of co-localization appear white in overlay images. Scale bar = 10 µm.

***Individual distribution patterns of YFP-tagged Gn and Gc is consistent among different cell lines.*** Besides Chinese hamster ovary cells (CHO-K1 cells), we also investigated the intracellular distribution of the chimeric PUUV glycoprotein constructs in two further cell lines relevant in the context of Hantavirus infection: Human embryonic kidney 293 cells (HEK293 cells) and African green monkey kidney epithelial cells (Vero E6 cells). Since Gn and Gc greatly differed in their association with the Golgi apparatus in CHO-K1 cells when expressed separately, we exclusively focused in this experiment on the cellular localization of Gn and Gc with respect to the Golgi apparatus. To this aim, HEK293 and Vero E6 cells were co-transfected with Golgi-Turq and the respective YFP-tagged glycoprotein construct and live-cell fluorescence microscopy was performed.

Both glycoprotein constructs exhibited the same distribution pattern in HEK293 and Vero E6 cells as observed in CHO-K1 cells (SI Fig. 3). Gn exhibited a negligible co-localization with the Golgi marker Golgi-Turq in both HEk293 and VeroE6 cells while Gc strongly co-localized with Golgi-Turq already when expressed individually. Thus, the different enrichment of Gn and Gc in the Golgi apparatus when expressed separately was consistently observed independent of the used cell model system.


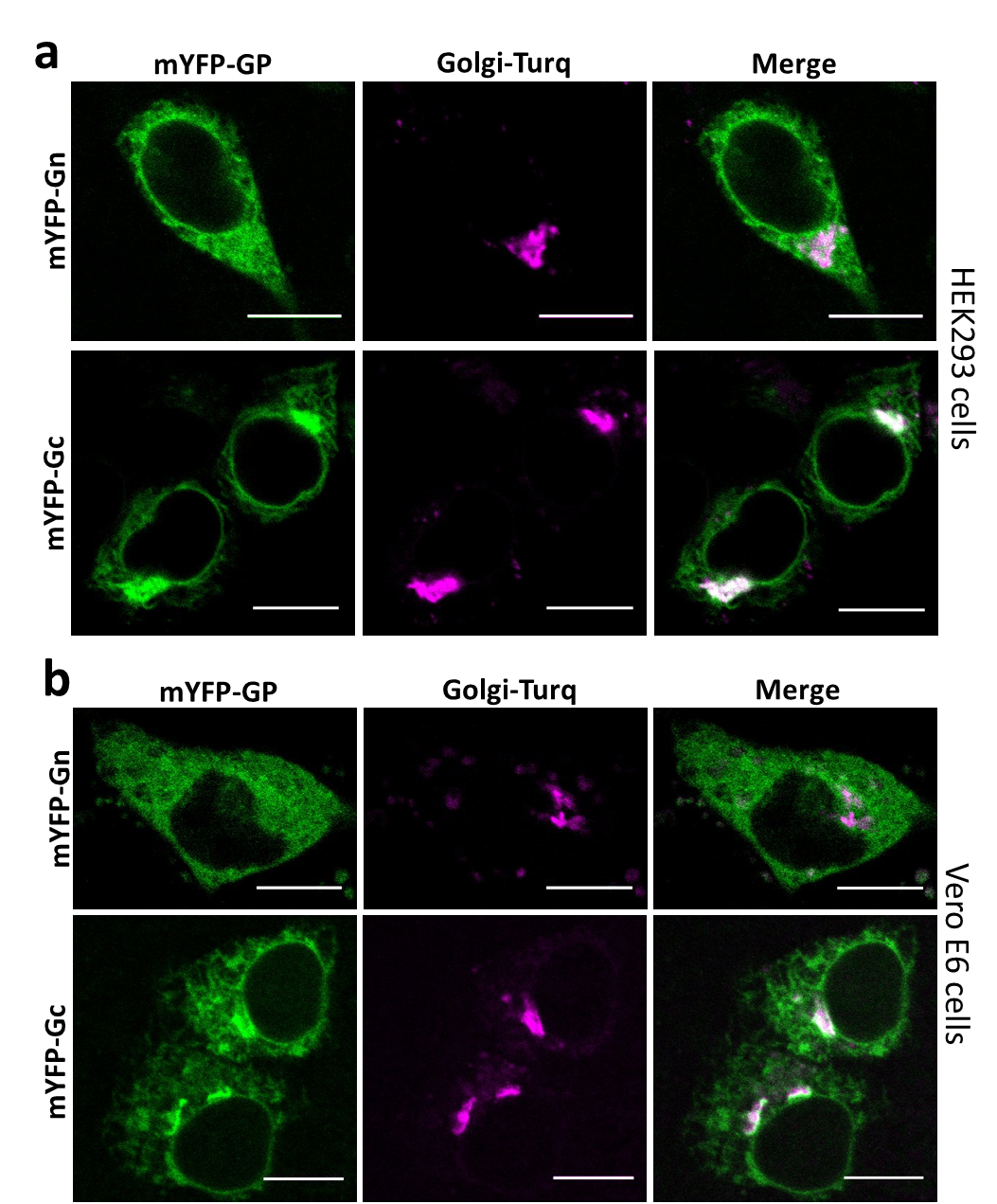


**SI Fig. 3: Intracellular localization of separately expressed PUUV chimeric glycoproteins in different cell lines.** (a) Human embryonic kidney 293 cells (HEK293 cells) or (b) African green monkey kidney epithelial cells (Vero E6 cells) were co-transfected with the YFP-labeled glycoproteins Gn or Gc (mYFP-GP, green) and the Golgi marker Golgi-Turq (purple). Fluorescence images represent equatorial slices obtained by confocal microscopy. High degrees of co-localization appear white in overlay images. Scale bar = 10 µm.

***Golgi localization of mYFP-Gc decreases significantly upon truncation of its CT.***


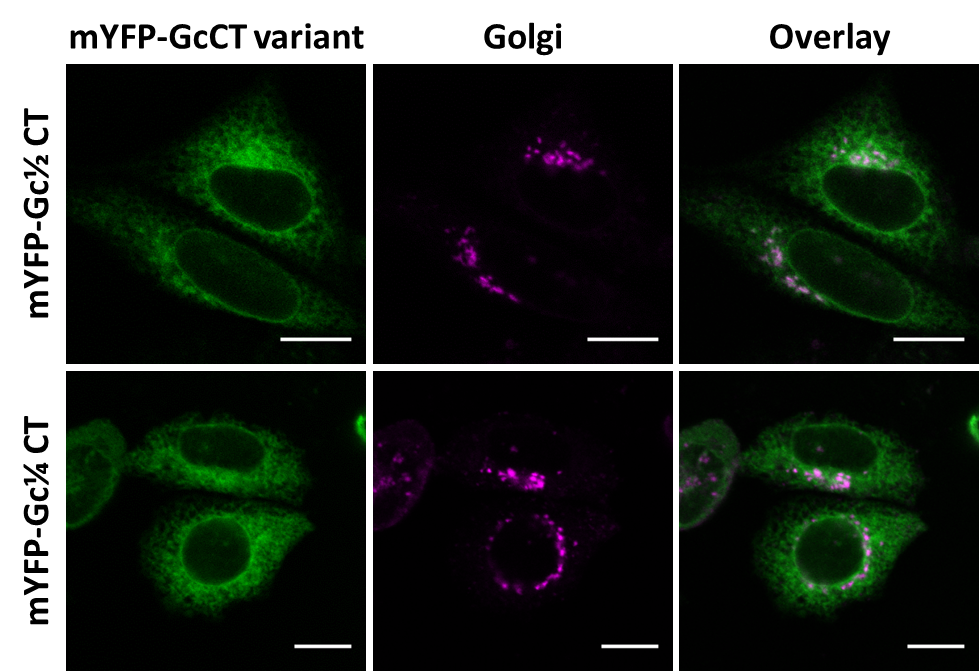


**SI Fig. 4: Colocalization of chimeric GcCT variants with the Golgi complex individually expressed in mammalian cells.** CHO-K1 cells were co-transfected with the mYFP-labeled glycoprotein variants mYFP-Gc½CT or mYFP-Gc¼CT and the Golgi maker Golgi-Turq (purple). Fluorescence images represent equatorial slices obtained by confocal microscopy 24h post transfection. High degrees of co-localization appear white in overlay images. Scale bar = 10 µm.

***mYFP-Gn is not subjected to autophagic degradation.*** Hussein et al.^2^ reported that Gn of the New World Sin Nombre Virus (SNV) is degraded via the autophagy lysosome pathway and that activity of the autophagy machinery is crucial for SNV replication. Thus, an explanation for the observed exclusion of mYFP-Gn from the Golgi region could be its degradation by the autophagy-lysosome pathway when transfected alone, which is prevented upon Turq-Gc co-expression. To test this hypothesis, we co-expressed mYFP-Gn with an LCP-3 autophagosome marker and assessed the fluorescence intensities in selected regions of interest (ROI) to determine co-localization of mYFP-Gn with autophagosomes (SI Fig. 5). mYFP-Gn did not co-localize with autophagosomes and even appeared to be excluded from these regions (SI Fig. 5). Moreover, we investigated the intracellular localization of mYFP-Gn following treatment with two different autophagy inhibitors: 3-methyladenine (3-MA) and LY-294002 (Ly) (SI Fig. 5). Inhibition of autophagy lysosome pathway did not alter the intracellular distribution pattern of mYFP-Gn and it remained in the ER as observed in absence of inhibitors (compare: Fig. 3a with SI Fig. 5). Based on these results, we conclude that PUUV Gn is not subject to autophagic degradation.


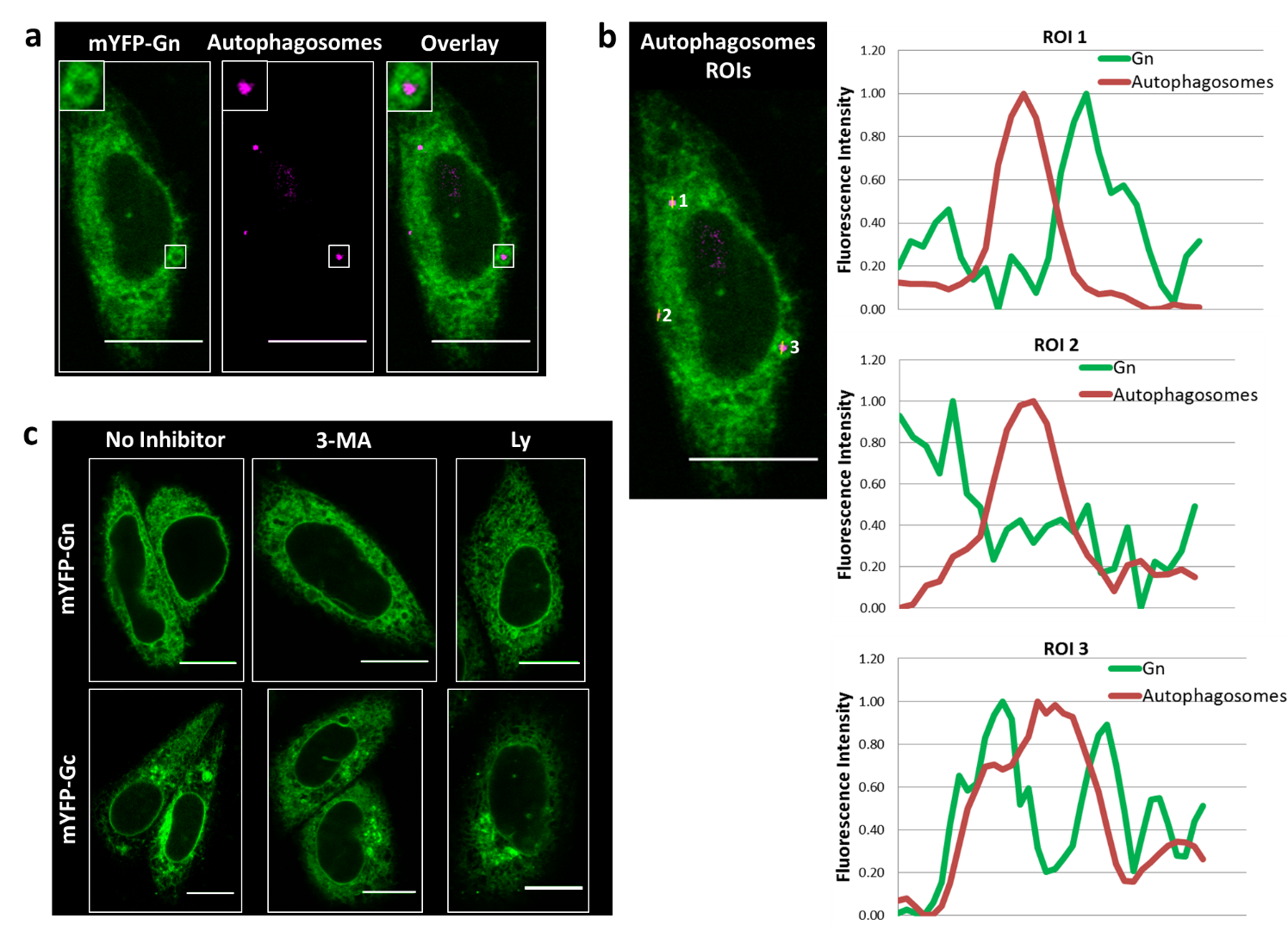


**SI Fig. 5: Involvement of mYFP-Gn in autophagy-lysosomal pathway.** (a) Co-transfection of mYFP-Gn (green) and the autophagosome marker LC3-RFP (red). Insets show magnifications of the boxed regions. Scale bar = 10 µm. (b) Measurement of fluorescence intensities of mYFP-Gn and LC3-RFP in selected regions of interest (ROIs). High signal intensity LC3-RFP spots, representing labeled autophagosomes, were selected as ROIs (marked as 1,2,3) and the fluorescence signal of both labeled proteins, mYFP-Gn and LC3-RFP, was assessed using ImageJ. A similar pattern of the fluorescence intensity would indicate correlation of the signal intensities and thus co-localization of mYFP-Gn with LC3-RFP (autophagosomes). However, the assessed fluorescence intensities are almost mutually exclusive, implying an exclusion of mYFP-Gn or no detectable mYFP-Gn in autophagosomes. (c) Intracellular distribution of mYFP-Gn and mYFP-Gc with and without the addition of autophagy inhibitors 3-methyladenine (3-MA) and LY-294002 (Ly). No difference in the distribution pattern of both glycoprotein constructs could be detected upon treatment with the respective autophagy inhibitors. Scale bar = 10 μm.

***The GnCT may play a crucial role in Gn homo oligomerization.*** Besides the original glycoprotein constructs, we also investigated the CT mutants mYFP-Gn_GcCT and mYFP-Gc_ΔCT via FAIM and N&B to scrutinize the role of the CTs in the oligomerization process. mYFP-Gn_GcCT displayed a higher anisotropy value than mYFP-Gn (SI Fig. 6a: mYFP-Gn vs. mYFP-Gn_GcCT). In contrast, elimination of the complete GcCT from mYFP-Gc resulted only in a weak increase of the anisotropy of mYFP-Gc_ΔCT compared to mYFP-Gc (SI Fig. 6a: mYFP-Gc vs. mYFP-Gc_ΔCT). Further, N&B analysis revealed that mYFP-Gc_ΔCT is present as mixture of monomers and dimers as mYFP-Gc, while mYFP-Gn_GcCT assembles probably almost exclusively into dimers (SI Fig. 6b). Thus, mYFP-Gn_GcCT did not assemble into tetramers anymore as observed for mYFP-Gn. Since deletion of the GcCT negligibly affected the FAIM or the N&B readout, we conclude that GcCT is not responsible for protein oligomerization, while the GnCT probably plays a determinant role in Gn-Gn interactions.


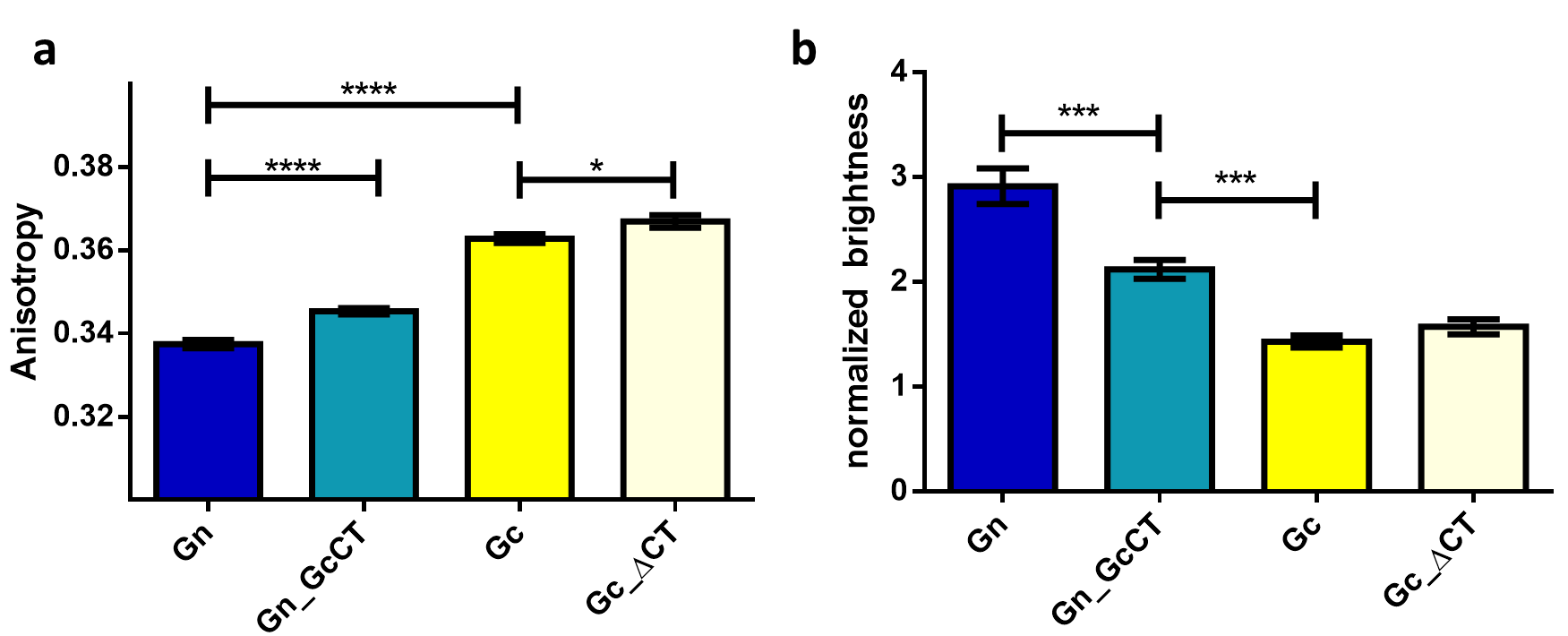


**SI Fig. 6: Oligomerization of PUUV glycoprotein chimera expressed in living CHO-K1 cells studied via fluorescence anisotropy and brightness mapping.** (a) CHO-K1 cells were transfected with fluorescent YFP constructs of Gn, Gc, Gc_ΔCT, and Gn_GcCT, and investigated by FAIM. Bars show the mean of single cell values of the YFP anisotropy of 52 cells expressing mYFP-Gn, 85 cells expressing mYFP-Gn_GcCT, 96 cells expressing mYFP-Gc, and 64 cells expressing mYFP-Gc_ΔCT from at least four independent experiments per specimen. Error bars show the standard error of the mean by Student’s test for unpaired data. The significance is displayed by asterisks: P≤0.0001 (****), P≤0.05 (*). (b) Brightness normalized using the corresponding monomer value extracted from the analysis of mYFP-ER or mYFP-Golgi (see main text for details). For each cell, an ROI enclosing the ER or the Golgi was defined and analyzed. The graph shows the mean values of the normalized brightness measured over 25-40 cells transfected with YFP-tagged fusion proteins from at least three independent experiments. Error bars represent the SEM. The asterisks denote p≤0.001 (***) as defined by MATLAB’s ttest2 function for unpaired data.

***Controls and additional information for N&B analysis.***

**SI Fig. 7: Examples of ROI selection for N&B analysis.** CHO-K1 cells were either co-transfected with mYFP-labeled Gn (A1) and ER-mCherry (A2) or with mYFP-labeled Gc (B1) and Golgi-mCherry (B2). The overlay of the two channels is shown (A3, B3). For each cell, an ROI enclosing the area marked by the highest-intensity pixels of the red channel was manually defined. Scale bar = 5 µm.


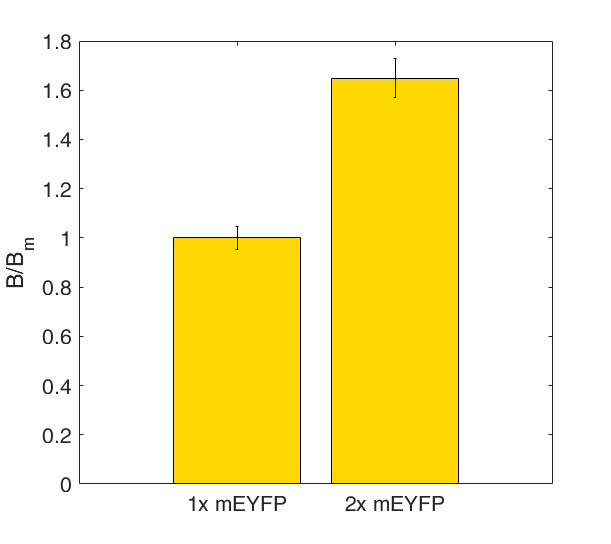


**SI Fig. 8: Normalized average mYFP monomer and dimer controls.** CHO-K1 cells were transfected with plasmids encoding a monomeric or a dimeric EYFP (1x mEYFP and 2x mEYFP respectively). Measurements were carried out on 34 (1x mEYFP) and 38 (2x mEYFP) cells in 4 separate experiments. Brightness values were normalized with respect to the value of the monomer, but do not yet take into consideration the maturation probability pm (see main text, Materials and Methods). Normalized brightness values shown in Fig. 5 are normalized with respect to the monomers and corrected for pm (i.e. a monomer has an expected value of 1, a dimer a value of 2 and so on). Error bars show the SEM.

**Supplementary Tables**

**SI Table 1: Statistics for bright detail similarity analysis.** The table displays the statistical significance between all analyzed samples, assessed by Student’s test for unpaired data, corresponding to Fig. 4h. Asterisks denote: p≤0.0001 (****), p≤0.001 (***), p≤0.01 (**), p≤0.05 (*), p>0.05 (ns). Black numbers indicated specific p-values. White numbers on black background show mean values of the respective sample.

| **BDS** | **mYFP-Gc** | **mYFP-Gc_½ CT** | **mYFP-Gc_¼ CT** | **mYFP-Gc_∆CT** | **mYFP-Gn** | **mYFP-Gn_GcCT** | **Golgi-YFP** |
| --- | --- | --- | --- | --- | --- | --- | --- |
| **mYFP-Gc** | **1.104** | **ns** | **ns** | ***** | ***** | ***** | ****** |
| **mYFP-Gc_½ CT** | **0.152** | **0.9352** | **ns** | ***** | **ns** | **ns** | ****** |
| **mYFP-Gc_¼ CT** | **0.070** | **0.444** | **0.875** | ****** | **ns** | **ns** | ****** |
| **mYFP-Gc_∆CT** | **0.015** | **0.045** | **0.003** | **0.746** | ***** | ******* | ****** |
| **mYFP-Gn** | **0.038** | **0.351** | **0.834** | **0.036** | **0.865** | **ns** | ******* |
| **mYFP-Gn_GcCT** | **0.034** | **0.354** | **0.950** | **0.001** | **0.835** | **0.873** | ******* |
| **Golgi-YFP** | **0.008** | **0.001** | **0.002** | **0.001** | **0.001** | **0.0003** | **1.671** |

**SI Table 2: Statistics for YFP intensity analysis.** The table displays the statistical significance between all analyzed samples, assessed by Student’s test for unpaired data, corresponding to Fig. 4i. Asterisks denote: p≤0.0001 (****), p≤0.001 (***), p≤0.01 (**), p≤0.05 (*), p>0.05 (ns). Black numbers indicated specific p-values. White numbers on black background show mean values of the respective sample.

| **YFP expression** | **mYFP-Gc** | **mYFP-Gc_½ CT** | **mYFP-Gc_¼ CT** | **mYFP-Gc_∆CT** | **mYFP-Gn** | **mYFP-Gn_GcCT** | **Golgi-YFP** |
| --- | --- | --- | --- | --- | --- | --- | --- |
| **mYFP-Gc** | **264538** | **ns** | **ns** | ****** | ****** | ***** | **ns** |
| **mYFP-Gc_½ CT** | **0.4424** | **241271** | **ns** | ****** | ******** | ****** | ***** |
| **mYFP-Gc_¼ CT** | **0.6571** | **0.0554** | **280739** | ****** | ******** | ****** | **ns** |
| **mYFP-Gc_∆CT** | **0.0052** | **0.0028** | **0.0031** | **131752** | ***** | **ns** | ******* |
| **mYFP-Gn** | **0.0013** | **<0.0001** | **<0.0001** | **0.0114** | **59921** | ****** | ******* |
| **mYFP-Gn_GcCT** | **0.0148** | **0.0059** | **0.0067** | **0.6979** | **0.0072** | **141073** | ****** |
| **Golgi-YFP** | **0.0658** | **0.0217** | **0.1324** | **0.0009** | **0.0008** | **0.0037** | **372369** |

**SI Table 3: Statistics for surface expression analysis.** The table displays the statistical significance between all analyzed samples, assessed by Student’s test for unpaired data, corresponding to Fig. 5. Asterisks denote: p≤0.0001 (****), p≤0.001 (***), p≤0.01 (**), p≤0.05 (*), p>0.05 (ns). Black numbers indicated specific p-values. White numbers on black background show mean values of the respective sample.

| **Surface YFP expression** | **GPI-mYFP** | **mYFP** | **mYFP-Gc** | **mYFP-Gc_½ CT** | **mYFP-Gc_¼ CT** | **mYFP-Gc_∆CT** | **mYFP-Gn** | **mYFP-Gn_GcCT** | **gp41-mYFP** | **∆1-mYFP** |
| --- | --- | --- | --- | --- | --- | --- | --- | --- | --- | --- |
| **GPI-mYFP** | **1** | ******** | ******** | ******** | ******** | ******** | ******** | ******* | ******* | ****** |
| **mYFP** | **<0.0001** | **0.00023** | ******* | ****** | ****** | ****** | ***** | **ns** | ***** | ***** |
| **mYFP-Gc** | **<0.0001** | **0.0004** | **0.02206** | ****** | ****** | ***** | **ns** | **ns** | **ns** | ***** |
| **mYFP-Gc_½ CT** | **<0.0001** | **0.0045** | **0.037** | **0.1083** | **ns** | ****** | ****** | ***** | **ns** | ***** |
| **mYFP-Gc_¼ CT** | **<0.0001** | **0.0056** | **0.0053** | **0.6524** | **0.1031** | ***** | ****** | ***** | **ns** | ***** |
| **mYFP-Gc_∆CT** | **<0.0001** | **0.0019** | **0.0123** | **0.0079** | **0.0108** | **0.03616** | ******* | **ns** | **ns** | ***** |
| **mYFP-Gn** | **<0.0001** | **0.0305** | **0.0723** | **0.0052** | **0.0067** | **0.0006** | **0.01449** | **ns** | **ns** | ***** |
| **mYFP-Gn_GcCT** | **0.0003** | **0.2999** | **0.8683** | **0.0316** | **0.0357** | **0.6186** | **0.6072** | **0.02551** | **ns** | ****** |
| **gp41-mYFP** | **0.0002** | **0.0355** | **0.0648** | **0.1396** | **0.1917** | **0.1124** | **0.0563** | **0.102** | **0.07554** | ***** |
| **∆1-mYFP** | **0.0045** | **0.0117** | **0.013** | **0.0197** | **0.0187** | **0.0141** | **0.0111** | **0.0059** | **0.011** | **0.3823** |

# **REFERENCES**

1. Goedhart, J. *et al.* Structure-guided evolution of cyan fluorescent proteins towards a quantum yield of 93%. *Nat. …* **3,** 751 (2012).

2. Hussein, I. T. M. *et al.* Autophagic clearance of Sin Nombre hantavirus glycoprotein Gn promotes virus replication in cells. *J. Virol.* **86,** 7520–9 (2012).
